# A quantitative systems pharmacology model of plasma kallikrein-kinin system dysregulation in hereditary angioedema

**DOI:** 10.1007/s10928-024-09919-6

**Published:** 2024-05-11

**Authors:** Dan Sexton, Hoa Q. Nguyen, Salomé Juethner, Haobin Luo, Zhiwei Zhang, Paul Jasper, Andy Z. X. Zhu

**Affiliations:** 1 Takeda Development Center Americas, Inc., Lexington, MA USA; 2 RES Group Inc., Needham, MA USA; 3 Preclinical and Translational Science Department, Takeda Pharmaceutical Company Limited, 35 Landsdowne Street, Cambridge, MA 02139 USA

**Keywords:** Quantitative systems pharmacology, Kallikrein-kinin system, Bradykinin, Hereditary angioedema, Lanadelumab

## Abstract

Hereditary angioedema (HAE) due to C1-inhibitor deficiency is a rare, debilitating, genetic disorder characterized by recurrent, unpredictable, attacks of edema. The clinical symptoms of HAE arise from excess bradykinin generation due to dysregulation of the plasma kallikrein-kinin system (KKS). A quantitative systems pharmacology (QSP) model that mechanistically describes the KKS and its role in HAE pathophysiology was developed based on HAE attacks being triggered by autoactivation of factor XII (FXII) to activated FXII (FXIIa), resulting in kallikrein production from prekallikrein. A base pharmacodynamic model was constructed and parameterized from literature data and ex vivo assays measuring inhibition of kallikrein activity in plasma of HAE patients or healthy volunteers who received lanadelumab. HAE attacks were simulated using a virtual patient population, with attacks recorded when systemic bradykinin levels exceeded 20 pM. The model was validated by comparing the simulations to observations from lanadelumab and plasma-derived C1-inhibitor clinical trials. The model was then applied to analyze the impact of nonadherence to a daily oral preventive therapy; simulations showed a correlation between the number of missed doses per month and reduced drug effectiveness. The impact of reducing lanadelumab dosing frequency from 300 mg every 2 weeks (Q2W) to every 4 weeks (Q4W) was also examined and showed that while attack rates with Q4W dosing were substantially reduced, the extent of reduction was greater with Q2W dosing. Overall, the QSP model showed good agreement with clinical data and could be used for hypothesis testing and outcome predictions.

**Supplementary Information:**

The online version contains supplementary material available at 10.1007/s10928-024-09919-6.

## Introduction

Hereditary angioedema (HAE) is a rare, autosomal dominant genetic disorder caused by a deficiency in functional C1 esterase inhibitor (C1-INH) with an estimated prevalence of 1 in 50,000 people worldwide [1]. HAE is potentially life-threatening and is characterized by unpredictable recurrent attacks of swelling in the extremities, face, larynx, and mucosal tissues. Attacks are highly variable in their time of onset, frequency, severity, and duration [2].

The underlying cause of HAE is a mutation in the gene encoding C1-INH, *SERPING-1*, resulting in reduced levels (HAE type 1) or function (HAE type 2) of the C1-INH protein [3], a negative regulator of plasma kallikrein. The kallikrein-kinin system (KKS) may become aberrantly activated in response to a variety of triggers including stress, hormonal changes, injury, exertion, sun exposure, and infection [4]. These triggers, the exact biochemical identification of which remains to be elucidated, may cause autoactivation of factor XII (FXII) to its activated form (FXIIa), which in turn converts prekallikrein to plasma kallikrein (referred to herein as kallikrein). In the absence of C1-INH, kallikrein cleaves high molecular weight kininogen (HMWK) to produce bradykinin and cleaved HMWK (cHMWK). Bradykinin activates bradykinin receptors on the surface of endothelial cells to cause vasodilation and increased blood vessel permeability, leading to subcutaneous or submucosal swelling and pain [5, 6].

Various pharmacological therapies are approved for the treatment of HAE. Icatibant is a competitive antagonist of the bradykinin B2 receptor that is indicated for the on-demand treatment of attacks [7]. In adults, a single subcutaneous injection of 30 mg icatibant provided symptom relief regardless of HAE attack location [8]. Treatments for the prevention of attacks include the following: Cinryze®, a plasma-derived C1-INH replacement therapy administered intravenously at a fixed dose of 1000 U every 3 or 4 days [9]; Haegarda®, another form of plasma-derived C1-INH, administered subcutaneously as weight-adjusted doses of 60 U/kg every 3 or 4 days [10]; berotralstat, a kallikrein inhibitor that is administered once daily as an oral 150 mg capsule [11]; and lanadelumab, a monoclonal antibody inhibitor of activated kallikrein, indicated for prophylaxis with a 300 mg dose administered every 2 weeks (Q2W), although this can be reduced to every 4 weeks (Q4W) if patients are attack free for more than 6 months [12].

We developed a mechanistic quantitative systems pharmacology (QSP) model that integrates current understanding of the pathophysiology of HAE with molecular, preclinical, and clinical data on the pharmacokinetics (PK), pharmacodynamics (PD), and efficacy of HAE treatments that target the KKS. QSP has been applied across many diseases as a strategy to aid in clinical drug development and decision making, such as patient and dose selection [13]. The QSP model described herein was developed to understand the relative effectiveness of various HAE treatments in suppressing HAE attacks, and to explore the optimal dosing regimen of these treatments and the impact of nonadherence on treatment outcomes.

## Methods

### Model description

An overview of the process that was followed for development of the model is shown in Fig. S1; the numbered sections described below refer to the steps in the figure. Bradykinin levels were linked to the onset of HAE attacks by modeling attack events observed in patients with HAE. The QSP model assumes that all attack triggers lead to a systematic perturbation that activates the KKS (i.e. increases the circulating plasma concentration of FXIIa), which generates bradykinin above a previously reported threshold plasma concentration of 20 pM [14], resulting in an HAE attack.

The overall QSP model for HAE incorporates both pharmacological properties of the drug such as the PK characteristics of the drug of interest and the pharmacodynamic (inhibitory) properties of the drug, as well as the disease specific biology associated with HAE such as the bradykinin threshold level to induce an HAE attack (Fig. S1). The model was developed initially alongside data for lanadelumab and was then modified to incorporate data for fixed-dose C1-INH. Clinical data from patients with HAE who received these therapies were used to calibrate and validate the model.

To test the model for the untreated state, a comparison of steady-state protein levels predicted by the KKS model versus reported levels in healthy individuals and in patients with HAE during remission was performed.

### Model structure and parameterization

**Fig. 1:**
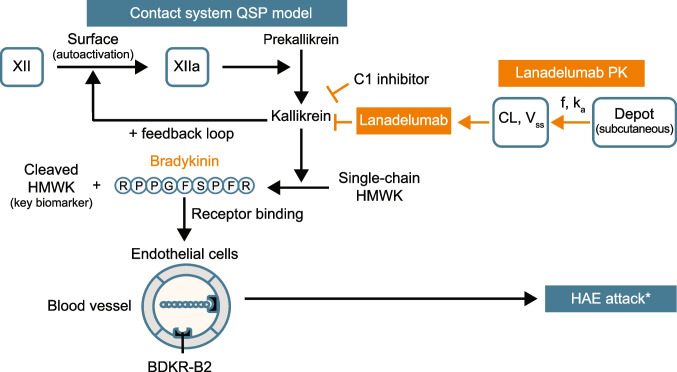
Overall scope of proposed QSP model. The model mechanistically links drug exposure to PD biomarkers and clinical outcome via the key biological pathway in HAE pathophysiology. *CL* clearance, HAE hereditary angioedema, *f* HAE attack frequency, *HMWK* high molecular weight kininogen, *k*_*a*_ rate of absorption after subcutaneous administration, *PD* pharmacodynamics*, PK* pharmacokinetics, *QSP* quantitative systems pharmacology, *V*_*ss*_ volume of disease at steady state. *HAE attack simulations were initiated by factor XIIa generation

As illustrated by the model diagram in Fig. 1 and model parameterization step in Fig. S1, a multistep process was used to parameterize and validate the PK, PD, and HAE disease clinical endpoint of the model. Clinical studies from which data were used to develop the model are listed in Table S1. The process is as follows:

1. *Pharmacokinetics.* The concentration–time profile of lanadelumab was simulated using a one-compartment model with values for first-order rate of absorption, volume of distribution, linear clearance, and interindividual variability obtained from a population PK analysis [15]. The pharmacokinetic parameters of fixed-dose C1-INH were obtained from the literature [16, 17].
2. Ex vivo* QSP model of the KKS system.* A two*-*step approach was used to establish the PD parameters of the key proteins in the KKS and their interactions. First, kallikrein activity from an ex vivo prekallikrein activation assay (Fig. S2) was modeled to verify the inhibitory effect of lanadelumab against kallikrein activity in plasma from healthy volunteers and patients with HAE who received lanadelumab in phase 1 studies [18, 19]. In this assay, the addition of FXIIa to plasma simulates activation of the KKS and is described mathematically by enzymatic reactions that form kallikrein in the presence of inhibitors (e.g. lanadelumab) (Table S2). Initial parameter values for the model were obtained from the literature (Table S3). The inhibition of kallikrein activity was calculated as:
  $$\%\;\mathrm{inhibition}=\left(1-\frac{{\text{KAL}}}{{{\text{KAL}}}_{{\text{control}}}}\right)*100{\%}$$
  where KAL_control_ was the simulated kallikrein activity in control samples after 2 min of FXIIa activation. Second, parameters developed from the ex vivo modeling described above were calibrated using plasma concentrations of KKS components from the literature, as described below.
3. In vivo* QSP model*. A biological map of the KKS and pharmacodynamic interactions assembled from literature is presented in Fig. S3, and details on the components and interactions are presented in the Supplement methods. The QSP model was constructed using the components and mathematical equations listed in Table S4 and Table S5, respectively, and the model parameters and model assumptions listed in Tables S6 and S7, respectively. The rate of change of each species was governed by its steady-state level, binding affinity, synthesis rate, and degradation rate. An additional parameter for the catalytic conversion rate was included for enzymatic conversion reactions. Basal levels of all species were obtained from the literature and were used to guide the calibration of the synthesis rate and catalytic rate for each species. Binding affinity of lanadelumab to kallikrein was obtained from the literature and verified against data from the fluorogenic assay. Binding affinities and degradation rates for other species were obtained from the literature, except for the degradation rate of cHMWK, which was calibrated to match reported levels in healthy subjects and patients with HAE. To be consistent with C1-INH deficiency in HAE, the synthesis rate of C1-INH for HAE patients was set to be lower than that of healthy individuals. An estimate of the kallikrein concentration generated during an HAE attack was obtained from the measurement of α-2-macroglobulin complexed with kallikrein [20]. Based on published physiological parameters [21], the vascular volume circulation time is approximately 100 s; therefore, for all soluble KKS components except bradykinin, the distribution clearance between proximal and vascular space was assumed to be the product of proximal volume and the inverse of circulation time. Bradykinin was assumed to have a faster exchange rate due to its smaller size compared to other species; thus, the distribution clearance was calibrated to capture the basal level in plasma. All other model parameters were the same for healthy subjects, HAE patients during remission, and HAE patients during an attack.
4. *Modelling HAE efficacy endpoint.* The link between PD biomarker (cHMWK level) and clinical endpoint (number of attacks) was modeled using attack events for a virtual cohort of patients with HAE (virtual patients differ in their PK profile, attack frequency, timing of triggering event, and attack severity). The propensity of a virtual patient to experience an attack was modeled by a distribution of varying attack frequency and severity. The trigger for an attack leading to autoactivation of the KKS was modeled as a random increase in the FXII autoactivation rate; the increase occurred according to simulated timing based on a Poisson process [22], with an assigned increase in autoactivation rate following a normal distribution, and based on input of the average attack frequency in patients. Attack occurrence and monthly attack rate were recorded when the bradykinin level exceeded 20 pM [14]. The baseline attack rate for each virtual patient was randomly sampled from a normal distribution with a mean of 3.5 attacks/month obtained from the HELP study (NCT02586805) [15]. An individual HAE attack can occur at any time and is independent of the time since the last attack, but attacks overall tend to occur at a constant rate [23]. Furthermore, the time between events is exponentially distributed with parameter λ (average number of events in a given time period); therefore, the time interval between events was sampled using the inverse cumulative density function method with λ = 3.5:
  $$\mathrm{Time\;interval}= \frac{-\mathrm{ ln}(1-{\text{p}})}{\uplambda } (0<{\text{p}}<1)$$

A duration of 12 h was used for all attacks in this study; this was based on the different phases of the pain score [24] for a patient with HAE experiencing an untreated attack. The pain stemming from swelling typically increases during the first 8–24-h and then gradually subsides over the next 24–72 h [24–26]. It was assumed that the onset of pain is triggered by an attack and the presence of pain represents the duration of the attack (i.e. the time during which the level of FXII autoactivation remained elevated). HAE attacks are assessed in clinical studies using patient-reported tools such as the visual analog score or with quality of life instruments [27], which are difficult to apply in modeling without a direct link to KKS components. HAE attacks are understood to be related to the level of bradykinin, and the increase in bradykinin during an attack is caused by an increase in KKS activation. The model thus correlates attack severity with the magnitude of increase in FXII autoactivation. We estimated the extent of the increase in FXII autoactivation rate in a population by calibrating the model so that levels of bradykinin and cHMWK from virtual HAE patients (n = 1000 in each case) during an attack match disease activity in the absence of prophylactic therapy.

(5) *Model validation*
  After parameterization, the model was validated using clinical data from patients treated with lanadelumab. First, the KKS model excluding attack events was run to verify that lanadelumab inhibits plasma kallikrein (i.e. suppresses the formation of cHMWK) by comparing the simulation output to results from a phase 1b study [19] (Fig. S4). Next, the KKS model including attack events was run for 1000 virtual patients to simulate variabilities in lanadelumab PK [15], attack frequency, and attack severity. Results from the simulations were compared with cHMWK levels and monthly attack rates observed in the HELP study [28].
(6) *Inclusion of the impact of C1-INH in the model*
  To evaluate the KKS model was updated to account for treatment with C1-INH concentrates using data for fixed-dose C1-INH. Reactions for the inhibition of kallikrein and FXIIa following the administration of C1-INH were added to the model, and system parameters for endogenous C1-INH were also applied. A one-compartment model with reported PK parameters [17] was used to describe the disposition of fixed-dose C1-INH. A new variable, “C1-INH activity”, was calculated as the ratio of total endogenous and administered C1-INH versus C1-INH in healthy controls. Baseline C1-INH activity was fixed at 31% of the healthy controls for all virtual patients, reflecting their measured baseline levels [9]. A baseline monthly attack rate of 4.23 was applied for virtual patients to replicate the baseline characteristics of patients from a clinical trial [29]. Other system parameters were the same as those for simulations of lanadelumab treatment. To validate the updated model, simulations were run for HAE patients treated with a single intravenous (IV) dose of 1000 U C1-INH or 1000 U C1-INH twice/week for 12 weeks to compare the predicted PK characteristics to those observed in clinical studies. In addition, attack rate predictions for patients treated with 1000 U C1-INH twice/week for 12 weeks were compared with reported attack suppression [29].
(7) *Application of the model*
  The validated model was used to evaluate the impact of treatment nonadherence and changes in dosing regimen on attack rates during treatment. Simulations for 1000 virtual patients with HAE were performed for the switch from 6 months of treatment with lanadelumab 300 mg Q2W to 6 months of treatment with 300 mg Q4W.

### Software

The QSP model was initially developed and calibrated using J2 Dynamic Modeling and Optimization Software (RES Group, Needham, MA, USA). J2 provides a high level, declarative input language suitable for describing complex mechanistic models. The algorithm implemented in J2 has been described previously [30–33]. The model was implemented using R software (version 3.5; R Foundation for Statistical Computing, Vienna, Austria). The R package “deSolve” (version 1.21) was used to run simulations. The R model code can be found in the Supplementary Material.

## Results

### Establishing the base system pharmacology model of the KKS in patients with HAE during remission

**Fig. 2:**
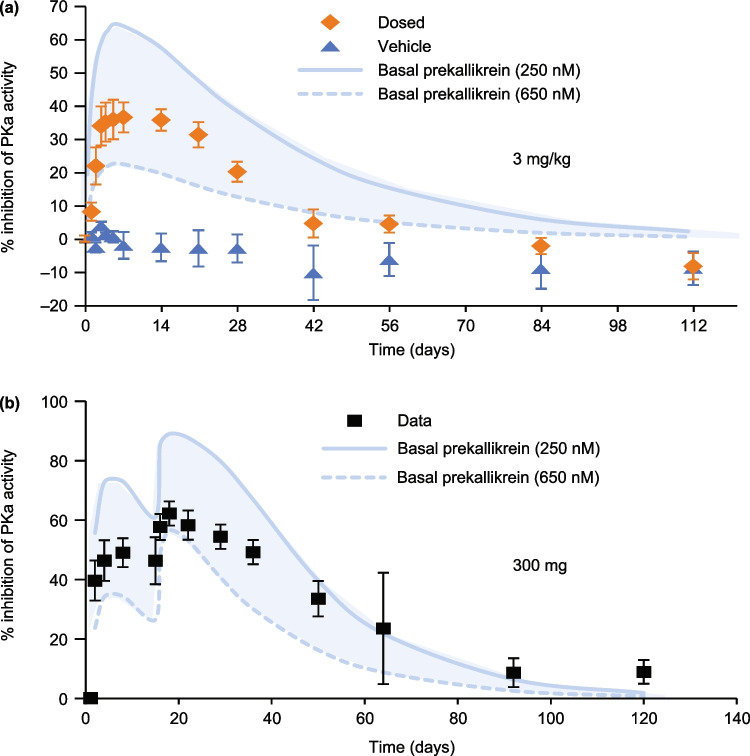
Ex vivo plasma prekallikrein activation assay. Citrated plasma was activated by the addition of FXIIa. The simulation output (shaded band) was compared to experimentally observed prekallikrein activity data from (**a**) healthy volunteers in phase 1a [18] and (**b**) HAE patients in phase 1b [19] studies at a dose of 3 mg/kg lanadelumab and 300 mg lanadelumab, respectively. The basal prekallikrein range corresponds to a previously reported range between 250 and 650 nM [36]. *FXIIa* activated factor XII, *HAE* hereditary angioedema, *PKa* prekallikrein

The ex vivo QSP model of the KKS was established using parameters from modeling of ex vivo plasma activation and fitted against published data. Data from the ex vivo fluorogenic assay of kallikrein activity using samples from healthy volunteers and HAE patients who received lanadelumab were compared with kallikrein activity results from clinical studies of lanadelumab [18, 19] (Fig. 2). Observed kallikrein inhibition data were well within the simulated results.

Using reference levels of all model species (Table 1), synthesis rates and catalytic rates in the KKS model were calibrated for agreement with reported values. A comparison of steady-state protein levels predicted by the KKS model versus reported levels in healthy subjects and in patients with HAE during remission is shown in Fig. S5.

**Table 1:** Plasma protein levels of species in the KKS compiled from literature data

Component	Plasma concentration (nM)		
	Healthy control	HAE patients	
		Remission	Acute attack
FXII	375 [41] (133–538) [42]	No change (type I) [43]	
FXIIa	0.019 (0.0116–0.023) [44]	0.03 (0.0175–0.0525) [45]	0.054 (0.035–0.1338) [45]
HMWK (120 KDa)	670 (375–2125) [41]	Decrease during attack (Type I) [43]	
Cleaved HMWK (110 KDa)	3.77 (2.88–4.67) [46]	12.35 (2.45–22.26) [46]	
PreKAL	365 (190–630) [36]	Decrease during attack (type I) [36]	
Free PreKAL%	18 (10–25) [47, 48]	18 (10–25) [47, 48]	
C1-INH	2400 (1400–3300) [41, 49]	240–720 [50]	144–432 [51]
BK	0.002 (0.0002–0.0071) [52]	0.0039 ± 0.0037 [14]	0.047 (0.015–0.077) [52]

*BK* bradykinin, *cHMWK* cleaved high molecular weight kininogen, *C1-INH* C1 esterase inhibitor, *FXII* factor XII, *FXIIa* activated FXII, *HAE* hereditary angioedema, *KKS* kallikrein-kinin system, *preKAL* prekallikrein

## Linking the KKS model to HAE attacks

**Fig. 3:**
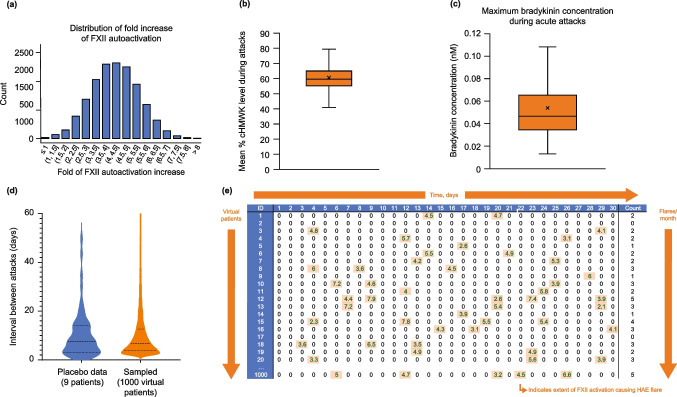
Illustration of acute attack event modeling. **a** Distribution of fold increase in autoactivation rate of FXII fitted to published bradykinin data [14]. **b** Comparison of % cHMWK obtained from the simulation of 1000 virtual patients to median levels observed in the HELP study (58%) [35], where % cHMWK is the percentage of cHMWK relative to the total of cHMWK and HMWK. **c** Calculated bradykinin level from the virtual population simulation. **d** The simulated time interval between attacks compared to the data from the same dose group. The attack occurrence events sampled from a Poisson distribution were consistent with the observed data. **e** A sample output of attack frequency and severity. The sample used 3.2 attacks per month [19]. The value within each attack event represents the fold increase in the autoactivation rate of FXII; nonzero values are highlighted. The X represents mean value and the box plots are min, Q1, median, Q3, and max. *cHMWK* cleaved high molecular weight kininogen, *conc* concentration, *HAE* hereditary angioedema, *HMWK* high molecular weight kininogen, *FXII* factor XII, *max* maximum, *min* minimum, *Q* quartile

Once the ex vivo QSP model of the KKS was established, the QSP model was developed further by linking biochemical changes in the KKS to HAE attacks. To trigger an attack, FXII autoactivation was increased. Different rates of FXII autoactivation were sampled from a normal distribution so that plasma bradykinin levels during an attack were in line with the reported range of 15–90 pM [34]. A mean (SD) 4.2-fold (1.2-fold normally distributed) increase in FXII autoactivation rate (Fig. 3a) was estimated to be required to match bradykinin levels during an attack.

Autoactivation of FXII and subsequent activation of plasma kallikrein results in a spike in bradykinin and cHMWK levels. Using the estimated extent of FXII autoactivation required for an attack as determined above, simulations were run for 1000 virtual patients over a 3-month baseline period. Based on the mean value of % cHMWK (difference between the peak and base) from each spike, a mean % cHMWK of 59% was derived during HAE attacks, similar to the reported level of 58% [35] (Fig. 3b).

The maximum plasma bradykinin [19] concentration during an attack as derived from simulations of virtual patient populations is shown in Fig. 3c; the mean and 25th/75th percentiles are well within the range of measured data [34]. In these simulations, a minimum threshold of 20 pM bradykinin in plasma was used as the criterion for an attack, representing the low end of the range of measured bradykinin levels for HAE patients during an attack.

The time intervals between successive attacks for 1000 virtual patients and the time intervals observed for the placebo group in a phase 1b study [19], both with an attack rate of 2.1 attacks/month, were well-aligned, indicating that the attack occurrences sampled from a Poisson distribution were consistent with the observed data (Fig. 3d). A sample output of attack frequency and severity for 1000 virtual patients over 30 days is shown in Fig. 3e. For each virtual patient, an attack rate of 3.2 attacks/month was used as input, and an attack event was generated via random sampling. The severity of each attack event represents the fold increase in the autoactivation rate of FXII.

## Model verification using data from a lanadelumab phase 3 study

**Fig. 4:**
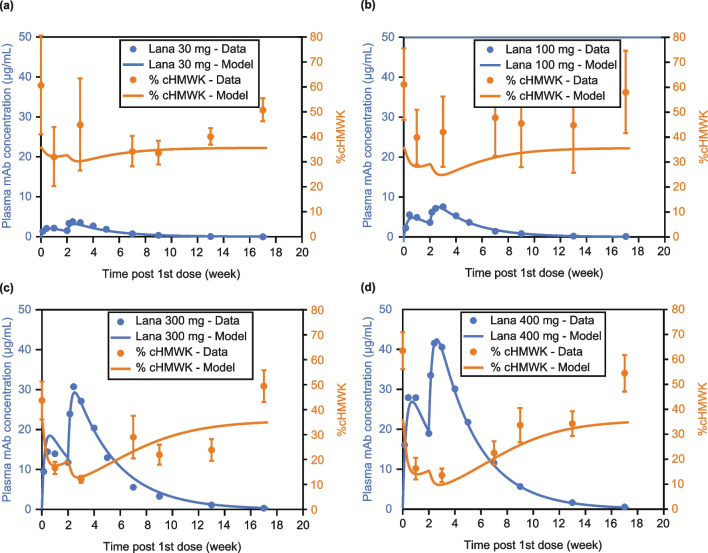
Comparison of cHMWK levels from a clinical study [19] to the simulation output from the QSP model for HAE patients treated with (**a**) 30 mg, (**b**) 100 mg, (**c**) 300 mg, and (**d**) 400 mg lanadelumab. The predicted PK profile is based on a one-compartment model. % cHMWK is the percentage of cHMWK relative to the sum of cHMWK + HMWK. *cHMWK* cleaved high molecular weight kininogen, *conc* concentration, *HAE* hereditary angioedema, *HMWK* high molecular weight kininogen, *Lana* lanadelumab, *mAb* monoclonal antibody, *PK* pharmacokinetics, *QSP* quantitative systems pharmacology, *w* week

Next, we investigated the ability of the model to predict biochemical changes in the KKS when a pharmacological intervention, lanadelumab, is administered. PK parameters for lanadelumab were obtained from the fitting of data from the lanadelumab clinical studies [19]. Figure 4 shows the relationship between lanadelumab dose and cHMWK levels from clinical data and the corresponding simulated profiles for virtual patients treated with 30, 100, 300, and 400 mg of lanadelumab. Results from the simulations agreed with clinical data showing an inverse correlation between drug concentration and cHMWK, and a notable reduction in cHMWK levels during the time of peak drug exposure (up to 8 weeks for the 300 mg and 400 mg dose groups).

**Fig. 5:**
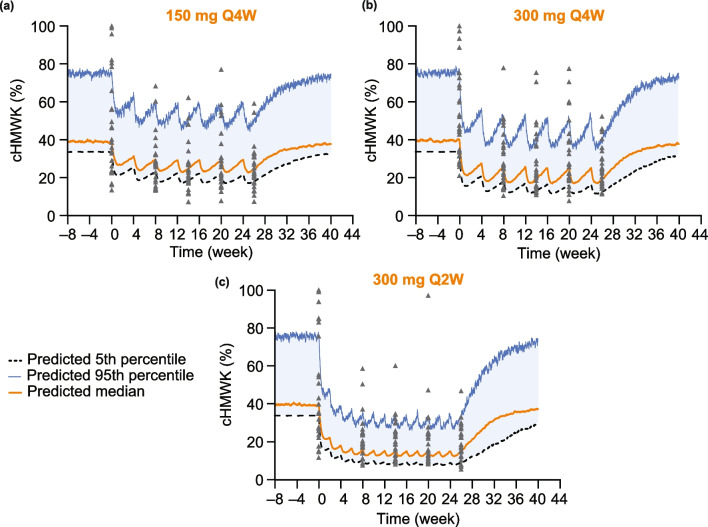
Comparison of cHMWK levels observed from clinical data to predicted levels from the QSP model simulation for virtual HAE patients treated with (**a**) 150 mg Q4W, (**b**) 300 mg Q4W, and (**c**) 300 mg Q2W lanadelumab. Symbols are data lines from simulation. The model prediction was made using 1000 virtual patients for each regimen group and the lanadelumab concentration was provided from the population PK model [15]. % cHMWK is the percentage of cHMWK relative to the total of cHMWK + HMWK. *cHMWK* cleaved high molecular weight kininogen, *HAE* hereditary angioedema, *HMWK* high molecular weight kininogen, *PK* pharmacokinetics, *Q2W* every 2 weeks, *Q4W* every 4 weeks, *QSP* quantitative systems pharmacology

**Fig. 6:**
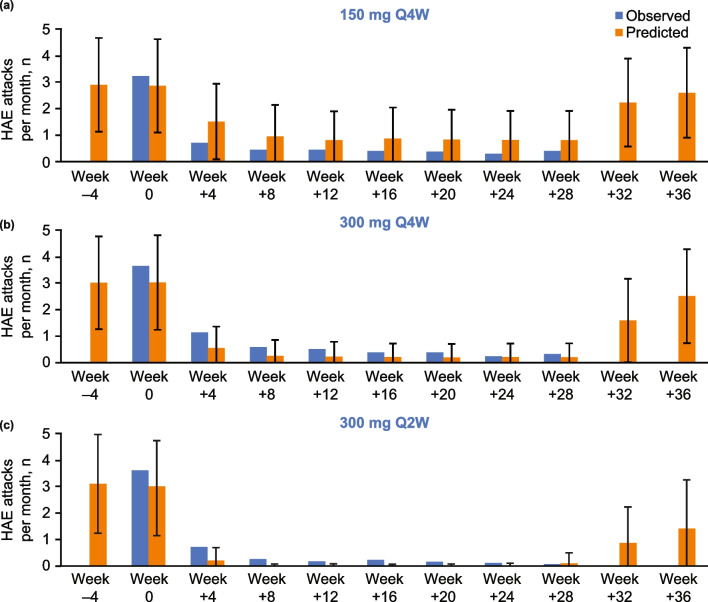
Comparison of HAE attack rates from observed clinical data and simulation output from the QSP model for a virtual cohort of 1000 HAE patients treated with (**a**) 150 mg Q4W, (**b**) 300 mg Q4W, and (**c**) 300 mg Q2W lanadelumab. The error bars represent 1 standard deviation. The clinical study had a duration of 28 weeks and no observed clinical data are available for weeks 32 and 36. *HAE* hereditary angioedema, *Q2W* every 2 weeks, *Q4W* every 4 weeks, *QSP* quantitative systems pharmacology

Furthermore, cHMWK levels from the HELP study [28] were compared with outputs from simulations of patients treated with lanadelumab 150 mg Q4W, 300 mg Q4W, and 300 mg Q2W (Fig. 5). In agreement with the clinical findings, the simulations showed a dose–response relationship in that cHMWK levels were reduced to a greater extent with higher dose (150 mg Q4W versus 300 mg Q4W) and more frequent dosing (300 mg Q4W versus 300 mg Q2W). Of note, the cHMWK level measured for some individual patients exceeded the 95th percentile envelope of the simulation; this may be attributed to the comparison data from individuals against a statistical output (percentile) from the simulation. The simulations also showed that all dose regimens are effective in suppressing the frequency of HAE attacks, in agreement with clinical observations (Fig. 6).

## Model validation with clinical data for fixed-dose C1-INH

**Fig. 7:**
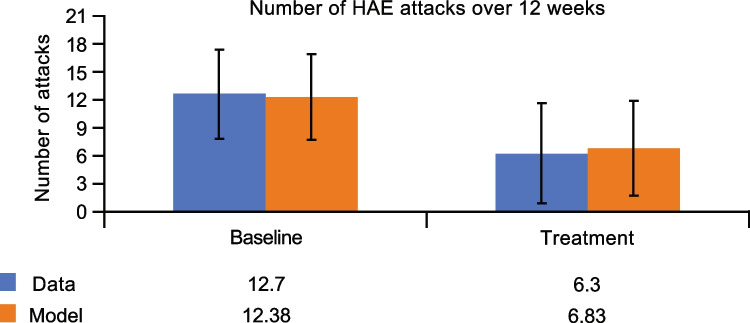
Simulation of attack frequency for fixed-dose C1-INH in comparison with clinical data [29]. *C1-INH* C1 esterase inhibitor, *HAE* hereditary angioedema

To generate further confidence in the KKS QSP model, the impact of fixed-dose C1-INH was investigated and compared to observed data. Without modifying or recalibrating the parameters of the above described KKS QSP model, simulated PK profiles for fixed-dose C1-INH were in agreement with mean concentrations of functional C1-INH protein in patients with HAE (Fig. S6) [29]. The same model was then used to run simulations for 1000 virtual patients following twice/week dosing of 1000 U C1-INH for 12 weeks. Attack rates from the model were consistent with attack rates observed in a clinical study (Fig. 7).

## Application of the model: effect of a missed dose

**Fig. 8:**
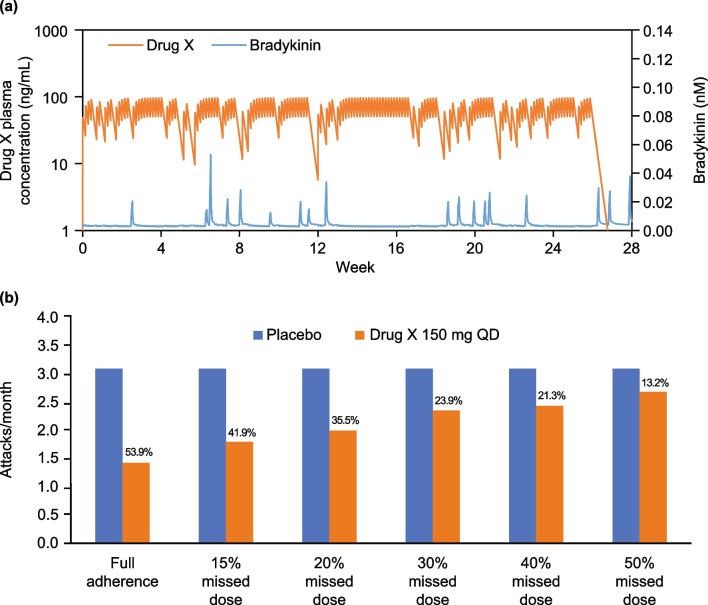
**a** Example pharmacokinetics of “drug X” 150 mg QD and bradykinin profiles with a nonadherence rate of 20%. **b** Simulated numbers of monthly attacks during treatment of “drug X” 150 mg QD with different nonadherence rates. Percentages represent reduction of attack rates compared with placebo. *QD* once daily

Nonadherence to preventive therapy was studied for a hypothetical oral drug with a shorter half-life (“drug X”) using simulations of virtual populations with an assumed average occurrence of nonadherence (random missing dose) of 20% (i.e. six missed doses in a 30-day month). Drug X was modeled as a once daily oral KAL inhibitor with a K_d_ of 0.44 nM and T1/2 of 19 h, which would result in an attack suppression of 54% during the first month after treatment. The model suggests that nonadherence for daily dosing of “drug X” could negatively impact the suppression of HAE attacks, as each missed dose would reduce drug levels, resulting in bradykinin peaks that exceed the 20 pM threshold for attacks (Fig. 8a). The monthly attack profiles showed that more missed doses resulted in increased occurrence of HAE attacks (Fig. 8b). Simulations showed that 6–8 days of randomly missed doses out of 4 weeks of daily dosing could translate into an approximately two-fold decline in efficacy. If ≥ 50% of doses are missed, efficacy becomes marginal.

## Application of the model: effect of lanadelumab down-titration

**Fig. 9:**
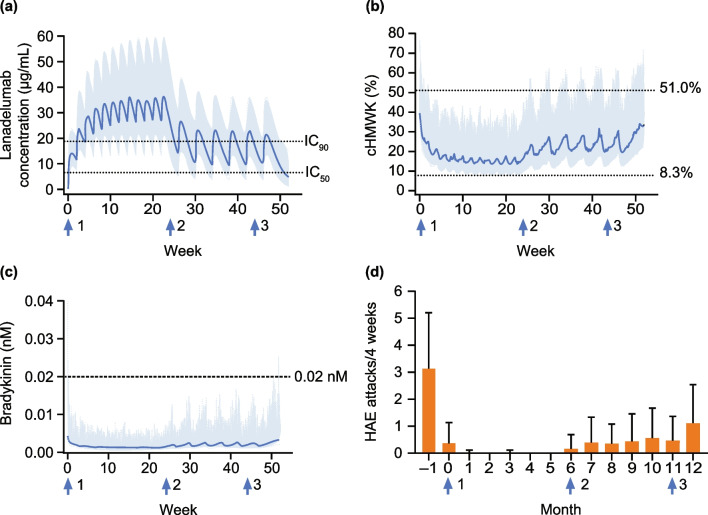
Simulations of lanadelumab 300 mg Q2W dosing for 6 months followed by Q4W dosing for 6 months. *HAE* hereditary angioedema, *Q2W* every 2 weeks, *Q4W* every 4 weeks

In simulations of virtual patients with a baseline attack rate of 3.5 attacks/month (similar to the mean attack rate among patients enrolled in the HELP study [28]), lanadelumab Q2W dosing resulted in mean minimum and maximum concentrations of lanadelumab above the 90% inhibitory concentration (IC_90_) of 18.8 µg/mL [15], mean cHMWK levels below 51%, and bradykinin levels below the 0.02 nM threshold for attacks (Fig. 9). After 6 months of Q2W treatment, the dosing frequency was reduced to Q4W, resulting in mean lanadelumab maximum concentrations, but not minimum concentrations, above IC_90_. However mean cHMWK levels remained below 51%, and bradykinin levels were less than 0.02 nM. Attack-free status, particularly in patients with high baseline attack rates (Fig. S7), was best achieved with Q2W dosing.

## Discussion

Model-informed drug development has been used to integrate information from diverse data sources to inform and support decision making during the drug development process. QSP is one approach that leverages thorough understandings of the disease pathology and the drug. A QSP model of the KKS was developed to quantitatively describe the dynamic interactions of the key components and biological pathways of the KKS and therapeutics that target the system. The model was linked to the frequency of attacks in HAE and provides a quantitative understanding of outcomes in clinical investigations of HAE therapeutics. Clinical drug development for rare diseases such as HAE is particularly challenging due to the small pool of patients available to participate in trials. QSP modeling is advantageous because it allows for outcome predictions in the absence of an additional clinical trial, thereby providing answers to questions quickly and accurately.

The workflow of the QSP model development began with a subset of the KKS using ex vivo data to explore and define the optimal scope of a model that can be reliably parameterized and verified. With all parameters initially estimated from literature data, the model for the ex vivo fluorescent assay was able to estimate observed inhibition of kallikrein activity [36]. This model was then expanded to include all key molecular elements in the KKS that are involved in HAE. KKS activation is localized on physiological surfaces and kallikrein is largely tethered to the endothelial cell surface through the prekallikrein–HMWK complex [37]. Consequently, the local (proximal space) concentration of kallikrein is simulated to approach the level of its precursor, prekallikrein (~ 500 nM) [38], with a simulated range between 40–400 nM during attacks depending on factors such as the volume of the local environment. While literature estimates for free, circulating kallikrein levels were unavailable, the model predicted a range from 0.2 to 1.5 nM. This low concentration of free kallikrein in the circulation could be due to the short half-life of kallikrein (~ 5 min) [39] and may contribute to attack localization, as opposed to systemic angioedema in HAE.

Alongside the modeling of HAE attack events, the QSP model was applied to verify the inhibitory effect of various therapeutics in HAE patients by comparing results from the simulations to biomarker data and attack rates observed in clinical trials. Reduction of cHMWK formation owing to kallikrein inhibition was sustained throughout the lanadelumab treatment period. The model demonstrated that the 300 mg Q2W regimen is optimal for achievement of therapeutic efficacy as indicated by the fastest and highest extent of cHMWK suppression over time, as well as the lowest average monthly attack rate compared with the other dosing regimens. Furthermore, the model showed that down-titration to Q4W dosing may still provide significant protection against HAE attacks, depending on the goals of treatment. The model supports the 300 mg Q2W regimen to achieve the therapeutic goal of being attack free; however, 300 mg Q4W dosing may be an appropriate option for some patients. Notably, a new steady state is reached approximately 70 days after the switch to Q4W dosing; thus, patients and their physicians should be mindful of this when monitoring the effectiveness of the new dose over time.

The key advantage of this QSP model is that it was constructed to cover the essential biological network of the KKS in HAE. Thus, the model allows for testing of its predictive capability against different treatment options. For example, the model was applied to analyze the impact of fixed-dose C1-INH using the same system parameters as the lanadelumab model, with only the PK component being updated for C1-INH; the primary endpoint (number of attacks) obtained from the model was in good agreement with reported results from clinical trials.

The model was also applied to investigate the potential impact of nonadherence to treatment with a theoretical orally administered daily inhibitor of plasma kallikrein, as a mean adherence rate of 80% (range ~ 65–90%) in the real world has been reported for daily oral therapy [40]. The model suggested that a nonadherence rate of 20% (i.e. five or six doses missed randomly in a month) results in a 35% increase in attack rate. Thus, with the short half-life of medications that must be administered daily, the apparent convenience of an oral dosing regimen is countered by a need to ensure strict adherence to treatment as a way of maintaining efficacy and cost effectiveness. On the other hand, the long half-life of lanadelumab results in maintenance of trough plasma concentrations above IC_90_ even with Q2W dosing, and less frequent dosing (13–26 doses/year) provides fewer opportunities to miss doses and experience breakthrough attacks.

QSP models mechanistically link different components in biological pathways to mathematically represent complex physiology and drug interactions. Application of QSP models can be challenging due to biological uncertainty and variability. To develop a model with a high level of validity, the goal was to improve understanding of the connection between mechanisms and outcomes. A virtual cohort of patients was generated to explore the impact of biological uncertainty and variability on response to therapies. Lastly, the model was challenged with approved medications targeting different elements of the KKS. The model may serve as a predictive platform for new drug development in the area of disease pathophysiology involving the KKS.

## Conclusions

The QSP model of KKS dysfunction in HAE shows promise as a useful predictive platform for new drug development in HAE. With rare diseases such as HAE, prospective trials that are meant to assess the impact of PK/PD changes on patient outcomes can be challenging to conduct. This model allows for hypothesis testing in the absence of an additional clinical trial, thereby facilitating drug discovery and development in HAE.

## Supplementary Information

Below is the link to the electronic supplementary material.

Supplementary file1 (R 9 KB)

Supplementary file2 (C 32 KB)

Supplementary file3 (R 4 KB)

Supplementary file4 (PDF 3460 KB)

Supplementary file5 (R 12 KB)

Supplementary file6 (R 6 KB)

Supplementary file7 (R 5 KB)

## Data Availability

The datasets, including the redacted study protocol, redacted statistical analysis plan, and individual participants’ data supporting the results reported in this article, will be made available within 3 months from initial request to researchers who provide a methodologically sound proposal. The data will be provided after its de-identification, in compliance with applicable privacy laws, data protection, and requirements for consent and anonymization.
