# Supplementary material for: A quantitative systems pharmacology model of plasma kallikrein-kinin system dysregulation in hereditary angioedema
Source: J Pharmacokinet Pharmacodyn. 2024 May 11;51(6):721–34. doi: 10.1007/s10928-024-09919-6 (PMC11579104; doi:10.1007/s10928-024-09919-6)
Supplement: Supplementary file 4 — Supplementary file4 (PDF 3460 KB) [file 10928_2024_9919_MOESM4_ESM.pdf]

A quantitative systems pharmacology model of plasma kallikrein-kinin system dysregulation in hereditary angioedema

Dan Sexton[1] • Hoa Q. Nguyen[1*] • Salomé Juethner[1] • Haobin Luo[2] • Zhiwei Zhang[2] • Paul Jasper[2] • Andy Z. X. Zhu[1]

[1]Takeda Development Center Americas, Inc., Lexington, MA, USA

[2]RES Group Inc., Needham, MA, USA

*Affiliation at the time of this study

Supplementary Information

List of Appendices

Method S1. Detailed description and model components.

Table S1 Summary of clinical trials from which data were incorporated into the QSP model.

Table S2 List of model equations used to describe *ex vivo* KKS activation in the fluorogenic assay.

Table S3 Inputs and parameters used in the fluorogenic assay model.

Table S4 List of molecular species in the KKS model.

Table S5 List of governing mathematical equations of the KKS model.

Table S6 List of model parameters.

Table S7 Model assumptions.

Fig. S1 Overview of the process for development of the HAE QSP model.

Fig. S2 Ex vivo assay measuring the inhibition of proteolytic activity of kallikrein by lanadelumab.

Fig. S3 Map of the KKS in HAE and pharmacodynamic interactions.

Fig. S4 Inhibition of plasma kallikrein by lanadelumab.

Fig. S5 Comparison of steady-state protein levels predicted by the KKS model to data from the literature.

Fig. S6 Simulated PK profiles were compared with mean concentrations of functional C1-INH protein in HAE patients treated with (a) a single IV dose of 1000 IU C1-INH or (b) 1000 IU C1-INH twice/week for 12 weeks.

Fig. S7 Predicted impact of down-titration for patients with high baseline attack rate (6 attacks/month). (a) Bradykinin levels and (b) attack rate with lanadelumab Q2W dosing for 6 months followed by Q4W dosing for 6 months.

List of abbreviations

BDKR-B2	B2 receptor of bradykinin receptor family
BK	Bradykinin
C1-INH	C1-esterase inhibitor
C1q	Complement component 1
gC1q-R	Cofactors complement protein C1q
CK1	Cytokeratin 1
cHMWK	Cleaved high molecular weight kininogen
% cHMWK	Percentage of cleaved high molecular weight kininogen relative to the total of cleaved high molecular weight kininogen and high molecular weight kininogen
FXII	Factor XII
FXIIa	Activated factor XII
HAE	Hereditary angioedema
HMWK	High molecular weight kininogen
KAL	Kallikrein
k_{cat}	Turnover number
K_d	Binding affinity
KKS	Kallikrein-kinin system
K_m	Michaelis-Menten constant
K_{on}	Association constant
K_{off}	Dissociation constant
K_{syn}	Synthesis rate
PD	Pharmacodynamics
PK	Pharmacokinetics
PRCP	Prolylcarboxypeptidase
preKAL	Prekallikrein
Q2W	Every 2 weeks
Q4W	Every 4 weeks

QSP	Quantitative systems pharmacology
SC	Subcutaneous
uPAR	Urokinase plasminogen activating receptor
V_{medium}	Per endothelial cell-based vascular volume
$V_{proximal}$	Per endothelial cell-based proximal space volume

Method S1. Detailed description of model components

Factor XII (FXII) is an 80 kDa glycosylated protein consisting of a single polypeptide chain and circulates in plasma as a zymogen with a concentration of 30 µg/mL (375 nM) in healthy individuals. Upon contact with anionic surfaces, and in the presence of Zn^{2+} ions, FXII undergoes a conformational rearrangement leading to autoactivation or cleavage by kallikrein to generate FXIIa (the activated form of FXII).

Prekallikrein is an 86 kDa glycoprotein consisting of a single polypeptide chain that circulates in plasma as a zymogen at a median concentration of 31 µg/mL (365 nM) in healthy individuals [1], it is estimated that 75% of prekallikrein is bound to high molecular weight kininogen (HMWK) [2]. Prekallikrein binds to endothelial cells, platelets, and granulocytes in a Zn^{2+}- dependent interaction via the prekallikrein–HMWK complex [3]. It is cleaved by FXIIa to form kallikrein, the two-chain enzyme kallikrein. Prolylcarboxypeptidase (PRCP) has been identified as an endothelial cell activator of prekallikrein to kallikrein [4].

HMWK is a 120 kDa non-enzymatic glycoprotein with a plasma concentration of 80 µg/mL (670 nM) in healthy individuals. HMWK circulates in plasma both in a free or complexed form (with prekallikrein or kallikrein). The binding affinities of HMWK to prekallikrein and kallikrein are similar (K_d of 12 nM and 15 nM, respectively [5]).

Assembly of the kinin-kallikrein contact factor proteins on cell surfaces is mediated via urokinase plasminogen activating receptor (uPAR), and cofactors complement protein C1q (gC1q-R) and cytokeratin 1 (CK1). On the surface of endothelial cells, gC1q-R (with elevated levels of Zn^{+2} ions released from endothelial cells and activated platelets) is primarily responsible for assembly and activation of the FXII-HMWK-prekallikrein complex, as previously demonstrated using surface plasmon resonance measurements showing that HMWK

has the greatest affinity to gC1q-R (0.8 nM), followed by CK1 (15 nM) and then uPAR (2.3 μM) [6]. Other studies suggested that FXII and HMWK compete with each other for binding to gC1q-R [7]. gC1q-R forms a multiprotein complex with uPAR and CK1, and the complex can bind to FXII [8]. In addition, the complex of uPAR with CK1 was shown to bind HMWK, resulting in prekallikrein activation at the endothelial cell surface [9].

The number of receptors, cofactors, and their complexes on endothelial cells has been reported. Among them, gC1q-R is the most abundant with over 1 million/cell [10], while uPAR (250,000/cell [11]) and CK1 (72,000/cell [9]) are expressed at lower levels. As the gC1q-R/CK1 complex preferentially binds HMWK, and FXII binds primarily to uPAR within the CK1-uPAR complex [12], it was assumed that CK1, with its lower expression, is the limiting factor for formation of the receptor complex in the activation of the surface KKS. The apparent affinity between FXII and HMWK to binding sites on human umbilical vein endothelial cells was reported to be 144 nM and 7–52 nM, respectively [7, 6].

Excessive bradykinin causes an increase in blood vessel permeability, which allows fluid to pass through the blood vessel walls, causing subcutaneous or submucosal swelling. The cleavage of HMWK by kallikrein produces a two-chain cleaved HMWK (cHMWK) and the bradykinin peptide. Bradykinin has a short half-life (less than 30 seconds in the blood of most species [13]) and strong affinity for the cell surface bradykinin B2 receptor (0.5 nM [14]). These properties make it challenging to obtain reliable measurements of bradykinin levels. However, cHMWK has been readily measured in clinical trials and serves as a valuable biomarker. Based on a saturation analysis of bradykinin binding to different human and rat bradykinin B2 receptors [14], we estimated the receptor number to be approximately 100,000/cell.

Table S1. Summary of clinical trials from which data were incorporated into the QSP model

Therapeutic	Phase	Study number	Title	Participants	Dosing	Reference
Lanadelumab	1a	NCT01923207	A Single Increasing Dose Study to Assess Safety and Tolerability of DX-2930 in Healthy Subjects	N = 32 healthy subjects	Single dose of placebo, 0.1, 0.3, 1.0, or 3.0 mg/kg lanadelumab	Chyung et al [15]
Lanadelumab	1b	NCT02093923	A Double-Blind, Multiple Ascending Dose Study to Assess Safety, Tolerability and Pharmacokinetics of DX-2930 in Hereditary Angioedema Participants	N = 37 patients with HAE type	Placebo or two doses of 30, 100, 300, 400 mg lanadelumab administered 14 days apart	Banerji et al [16]
Lanadelumab	3	NCT02586805	Efficacy and Safety Study of DX-2930 to Prevent Acute Angioedema Attacks in Patients With Type I and Type II HAE (HELP)	N = 125 patients with HAE type	Placebo or 150 mg Q4W, 300 mg Q4W, 300 mg Q2W lanadelumab for 26 weeks	Banerji et al [17]
Lanadelumab	3	NCT02741596	Long-term Safety and Efficacy Study of DX-2930 (SHP643) to Prevent Acute Angioedema Attacks in Patients With Type I and Type II HAE (HELP OLE)	N = 212 patients with HAE type	300 mg Q2W lanadelumab for up to 132 weeks	Banerji et al [18]
Cinryze®	3	NCT00289211	A Double-blind, Placebo-Controlled, Clinical Study to Investigate the Efficacy and Safety of Purified C1 Esterase Inhibitor (Human) for the Treatment of HAE in Acute Attacks	N = 68 patients with HAE	Placebo or 1000 U C1-INH (up to two injections)	Zuraw et al [19]
Cinryze®	3	NCT01005888	A Double-blind, Placebo-Controlled, Clinical Study to Investigate the Efficacy and Safety of Purified C1 Esterase Inhibitor (Human) as Prophylactic Treatment to Prevent HAE Attacks	N = 22 patients with HAE	Placebo or 1000 U C1-INH twice-weekly for 12 weeks	Zuraw et al [19]
Cinryze®	3	NCT00438815	Open-Label Safety/Efficacy Repeat Exposure Study of C1INH-nf (Human) in the Treatment of Acute HAE Attacks (CHANGE 2)	N = 113 patients with HAE	1000 U C1-INH as needed	Riedl et al [20]

Cinryze®	3	NCT00462709	Open-Label Use of C1INH-nf (Human) for the Prophylactic Treatment to Prevent HAE Attacks and as Treatment in Acute HAE Attacks (CHANGE 3)	N = 146 patients with HAE	1000 U C1-INH every 3 to 7 days	Shire [21]

C1-INH C1 esterase inhibitor, *HAE* hereditary angioedema, *Q2W* every 2 weeks, *Q4W* every 4 weeks, *QSP* quantitative systems pharmacology

Table S2 List of model equations used to describe *ex vivo* KKS activation in the fluorogenic assay

$\frac{d(preKAL)}{dt} = -\frac{kcat * FXIIa * preKAL}{K_m + preKAL}$
$\frac{d(KAL)}{dt} = \frac{kcat * FXIIa * preKAL}{K_m + preKAL} - (kon_{KAL-C1Inh} * KAL * C1Inh - koff_{KAL-C1Inh} * KAL_{C1Inh}) -(kon_{KAL-Lanadelumab} * KAL * Lanadelumab - koff_{KAL-Lanadelumab} * KAL\_Lanadelumab)$
$\frac{d(C1Inh)}{dt} = -(kon_{KAL-C1Inh} * KAL * C1Inh - koff_{KAL-C1Inh} * KAL\_C1Inh)$
$\frac{d(KAL\_C1Inh)}{dt} = (kon_{KAL-C1Inh} * KAL * C1Inh - koff_{KAL-C1Inh} * KAL\_C1Inh)$
$\frac{d(Lanadelumab)}{dt} = -(kon_{KAL-Lanadelumab} * KAL * Lanadelumab - koff_{KAL-Lanadelumab} * KAL\_Lanadelumab)$
$\frac{d(KAL\_Lanadelumab)}{dt} = (kon_{KAL-Lanadelumab} * KAL * Lanadelumab - koff_{KAL-Lanadelumab} * KAL\_Lanadelumab)$

C1-INH: C1 esterase inhibitor, *FXII* factor XII, *KKS* kallikrein-kinin system, *KAL* kallikrein, k_{cat} turnover number, K_m Michaelis-Menten constant, K_{off} dissociation constant, K_{on} association constant, *preKAL* prekallikrein

Table S3 Inputs and parameters used in the fluorogenic assay model

Input/Parameter	Explanation	Unit	Value used in model	Reference
preKAL	Basal prekallikrein level in plasma	nM	250–650	Madsen et al [1]
KAL	Basal kallikrein level in plasma	nM	0	Assumed negligible
C1Inh_normal	Basal plasma C1-INH level for normal population	nM	2400	Weidmann et al [22]
C1Inh_HAE	Basal plasma C1-INH level for HAE type 1 population	nM	600	Cicardi et al [23]
Kd_Lana_KAL	Binding affinity of lanadelumab to kallikrein	nM	0.12	Kenniston et al [24]
Kd_C1Inh_KAL	Binding affinity of C1-INH to kallikrein	nM	150	Harpel et al [25]
Km	K_m for activation of prekallikrein by FXIIa	uM	1.8	Tankersley and Finlayson [26]
kcat	k_{cat} for activation of prekallikrein by FXIIa	1/s	1.03	Tankersley and Finlayson [26]

C1-INH C1 esterase inhibitor, *FXIIa* activated factor XII, *HAE* hereditary angioedema, *KAL* kallikrein, k_{cat} turnover number, K_m Michaelis-Menten constant, *preKAL* prekallikrein

Table S4 List of molecular species in the KKS model

Variable names used in model code	Unit	Description
In vascular space		
BK_degraded	nM	Degraded bradykinin concentration
BK_in_plasma	nM	Bradykinin concentration
C1Inh_degraded	nM	Degraded C1-INH concentration
C1Inh_FXIIa_degraded	nM	Degraded C1-INH_FXIIa complex concentration
C1Inh_FXIIa_in_plasma	nM	C1-INH_FXIIa concentration
C1Inh_in_plasma	nM	C1-INH concentration
C1Inh_KAL_degraded	nM	Degraded C1-INH_kallikrein complex concentration
C1Inh_KAL_HMWK_degraded	nM	Degraded C1-INH_kallikrein_HMWK complex concentration
C1Inh_KAL_HMWK_in_plasma	nM	C1-INH_kallikrein_HMWK complex concentration
C1Inh_KAL_in_plasma	nM	C1-INH_kallikrein complex concentration
FXII_degraded	nM	Degraded FXII concentration
FXII_in_plasma	nM	FXII concentration
FXIIa_degraded	nM	Degraded FXIIa concentration
FXIIa_in_plasma	nM	FXIIa concentration
HK2Chain_degraded	nM	Degraded cHMWK concentration
HK2Chain_in_plasma	nM	cHMWK concentration
HMWK_degraded	nM	Degraded HMWK concentration
HMWK_in_plasma	nM	HMWK concentration
KAL_degraded	nM	Degraded kallikrein concentration
KAL_HK2Chain_in_plasma	nM	kallikrein_cHMWK complex concentration

KAL_HMWK_in_plasma	nM	kallikrein_HMWK concentration
KAL_in_plasma	nM	kallikrein concentration
Lanadelumab_in_plasma	nM	Lanadelumab concentration
Lanadelumab_KAL_HMWK_in_plasma	nM	Lanadelumab_kallikrein_HMWK complex concentration
Lanadelumab_KAL_in_plasma	nM	Lanadelumab_kallikrein complex concentration
preKAL_degraded	nM	Degraded prekallikrein concentration
preKAL_HMWK_in_plasma	nM	prekallikrein_HMWK complex concentration
preKAL_in_plasma	nM	prekallikrein concentration
In proximal space		
BK	nM	Bradykinin concentration
BDKRB2	number/cell	Number of bradykinin B2 receptors
BDKRB2_degraded	number/cell	Number of degraded surface bradykinin B2 receptors
BK_BDKRB2	number/cell	Number of bradykinin_bradykinin B2 receptor complexes
BK_BDKRB2_degraded	number/cell	Number of degraded surface bradykinin_bradykinin B2 receptor complexes
C1Inh	nM	C1-INH concentration
C1Inh_FXIIa	nM	C1-INH_FXIIa concentration
C1Inh_FXIIa_gC1qR	number/cell	Number of C1-INH_FXIIa_gC1q receptor complexes
C1Inh_FXIIa_gC1qR_degraded	number/cell	Number of degraded surface C1-INH_FXIIa_gC1q receptor complexes
C1Inh_KAL_HMWK	nM	C1-INH_kallikrein_HMWK concentration

C1Inh_KAL_HMWK_gC1qR	number/cell	Number of C1-INH_kallikrein_HMWK_gC1q receptor complexes
C1Inh_KAL_HMWK_gC1qR_degraded	number/cell	Number of degraded surface C1-INH_kallikrein_HMWK_gC1q receptor complexes
FXII	nM	FXII concentration
FXII_gC1qR	number/cell	Number of FXII_gC1q receptor complexes
FXII_gC1qR_degraded	number/cell	Number of degraded surface FXII_gC1q receptor complexes
FXIIa	nM	FXIIa concentration
FXIIa_gC1qR	number/cell	Number of FXIIa_gC1q receptor complexes
FXIIa_gC1qR_degraded	number/cell	Number of degraded surface FXIIa_gC1q receptor complexes
gC1qR	number/cell	Number of gC1q receptors
gC1qR_degraded	number/cell	Number of degraded surface gC1q receptors
KAL_HK2Chain	nM	kallikrein_cHMWK concentration
KAL_HK2Chain_gC1qR	number/cell	Number of kallikrein_cHMWK_gC1q receptor complexes
KAL_HK2Chain_gC1qR_degraded	number/cell	Number of degraded surface kallikrein_cHMWK_gC1q receptor complexes
KAL_HMWK	nM	kallikrein_HMWK concentration
KAL_HMWK_gC1qR	number/cell	Number of kallikrein_HMWK_gC1q receptor complexes
KAL_HMWK_gC1qR_degraded	number/cell	Number of degraded surface kallikrein_HMWK_gC1q receptor complexes
Lanadelumab	nM	Lanadelumab concentration

Lanadelumab_KAL_HMWK	nM	Lanadelumab_kallilkrein_HMWK concentration
Lanadelumab_KAL_HMWK_gC1qR	number/cell	Number of Lanadelumab_kallikreinL_HMWK_gC1q receptor complexes
Lanadelumab_KAL_HMWK_gC1qR_degraded	number/cell	Number of degraded surface Lanadelumab_kallikrein_HMWK_gC1q receptor complexes
preKAL_HMWK	nM	prekallikrein_HMWK concentration
preKAL_HMWK_gC1qR	number/cell	Number of prekallikrein_HMWK_gC1q receptor complexes
preKAL_HMWK_gC1qR_degraded	number/cell	Number of degraded surface prekallikrein_HMWK_gC1q receptor complexes

BDKR-B2 B2 receptor of bradykinin receptor family, *BK* bradykinin, *C1-INH* C1 esterase inhibitor, *C1q* complement component 1, *cHMWK* cleaved high molecular weight kininogen, *FXII* factor XII, *FXIIa* activated factor XII, *gC1q-R* cofactors complement protein C1q, *HK2* human kallikrein 2, *HMWK* high molecular weight kininogen, *KAL* kallikrein, *KKS* kallikrein-kinin system, *preKAL* prekallikrein

Table S5 List of governing mathematical equations of the KKS model

In vascular space	Equation number
$V_{medium}\frac{d\boldsymbol{BK\_degraded}}{dt} = V_{medium} \cdot kdeg_{BK} \cdot \boldsymbol{BK\_in\_plasma}$	**(E1)**
$V_{medium}\frac{d\boldsymbol{BK\_in\_plasma}}{dt} = -V_{medium} \cdot kdeg_{BK} \cdot \boldsymbol{BK\_in\_plasma} - (k12_{BK} \cdot \boldsymbol{BK\_in\_plasma} \cdot V_{medium} - k21_{BK} \cdot \boldsymbol{BK} \cdot V_{proximal})$	**(E2)**
$V_{medium}\frac{d\boldsymbol{C1Inh\_degraded}}{dt} = V_{medium} \cdot kdeg_{C1Inh} \cdot \boldsymbol{C1Inh\_in\_plasma}$	**(E3)**
$V_{medium}\frac{d\boldsymbol{C1Inh\_FXIIa\_degraded}}{dt} = V_{medium} \cdot kdeg\_Bound_{C1Inh} \cdot \boldsymbol{C1Inh\_FXIIa\_in\_plasma}$	**(E4)**
$V_{medium}\frac{d\boldsymbol{C1Inh\_FXIIa\_in\_plasma}}{dt} = V_{medium} \cdot (kon_{C1Inh\_FXIIa} \cdot \boldsymbol{C1Inh\_in\_plasma} \cdot \boldsymbol{FXIIa\_in\_plasma} - koff_{C1Inh\_FXIIa} \cdot \boldsymbol{C1Inh\_FXIIa\_in\_plasma}) - V_{medium} \cdot kdeg\_Bound_{C1In} \cdot \boldsymbol{C1Inh\_FXIIa\_in\_plasma} - (k12 \cdot \boldsymbol{C1Inh\_FXIIa\_in\_plasma} \cdot V_{medium} - k21 \cdot \boldsymbol{C1Inh\_FXIIa} \cdot V_{proximal})$	**(E5)**
$V_{medium}\frac{d\boldsymbol{C1Inh\_in\_plasma}}{dt} = flux\_C1Inh\_inj\_nmol\_per\_hr + V_{medium} \cdot ksyn_{C1Inh} - V_{medium} \cdot kdeg_{C1Inh} \cdot \boldsymbol{C1Inh\_in\_plasma} - V_{medium} \cdot (kon_{C1Inh\_KAL} \cdot \boldsymbol{C1Inh\_in\_plasma} \cdot \boldsymbol{KAL\_in\_plasma} - koff_{C1Inh\_KAL} \cdot \boldsymbol{C1Inh\_KAL\_in\_plasma}) - V_{medium} \cdot (kon_{C1Inh\_KAL} \cdot \boldsymbol{C1Inh\_in\_plasma} \cdot \boldsymbol{KAL\_HMWK\_in\_plasma} - koff_{C1Inh\_KAL} \cdot \boldsymbol{C1Inh\_KAL\_HMWK\_in\_plasma}) - V_{medium} \cdot (kon_{C1Inh\_FXIIa} \cdot \boldsymbol{C1Inh\_in\_plasma} \cdot \boldsymbol{FXIIa\_in\_plasma} - koff_{C1Inh\_FXIIa} \cdot \boldsymbol{C1Inh\_FXIIa\_in\_plasma}) - (k12 \cdot \boldsymbol{C1Inh\_in\_plasma} \cdot V_{medium} - k21 \cdot \boldsymbol{C1Inh} \cdot V_{proximal})$	**(E6)**

$$V_{medium}\frac{d\boldsymbol{C1Inh\_KAL\_degraded}}{dt} = V_{medium} \cdot kdeg\_Bound_{C1Inh} \cdot \boldsymbol{C1Inh\_KAL\_in\_plasma} \quad \textbf{(E7)}$$

$$V_{medium}\frac{d\boldsymbol{C1Inh\_KAL\_HMWK\_degraded}}{dt} = V_{medium} \cdot kdeg\_Bound_{C1Inh} \cdot \boldsymbol{C1Inh\_KAL\_HMWK\_in\_plasma} \quad \textbf{(E8)}$$

$$V_{medium}\frac{d\boldsymbol{C1Inh\_KAL\_HMWK\_in\_plasma}}{dt} = V_{medium} \cdot (kon_{C1Inh\_KAL} \cdot \boldsymbol{C1Inh\_in\_plasma} \cdot \boldsymbol{KAL\_HMWK\_in\_plasma} - koff_{C1Inh\_KAL} \cdot \boldsymbol{C1Inh\_KAL\_HMWK\_in\_plasma}) - V_{medium} \cdot kdeg\_Bound_{C1Inh} \cdot \boldsymbol{C1Inh\_KAL\_HMWK\_in\_plasma} - (k12 \cdot \boldsymbol{C1Inh\_KAL\_HMWK\_in\_plasma} \cdot V_{medium} - k21 \cdot \boldsymbol{C1Inh\_KAL\_HMWK} \cdot V_{proximal}) \quad \textbf{(E9)}$$

$$V_{medium}\frac{d\boldsymbol{C1Inh\_KAL\_in\_plasma}}{dt} = V_{medium} \cdot (kon_{C1Inh\_KAL} \cdot \boldsymbol{C1Inh\_in\_plasma} \cdot \boldsymbol{KAL\_in\_plasma} - koff_{C1Inh\_KAL} \cdot \boldsymbol{C1Inh\_KAL\_in\_plasma}) - V_{medium} \cdot kdeg\_Bound_{C1Inh} \cdot \boldsymbol{C1Inh\_KAL\_in\_plasma} \quad \textbf{(E10)}$$

$$V_{medium}\frac{d\boldsymbol{FXII\_degraded}}{dt} = V_{medium} \cdot kdeg_{FXII} \cdot \boldsymbol{FXII\_in\_plasma} \quad \textbf{(E11)}$$

$$V_{medium}\frac{d\boldsymbol{FXII\_in\_plasma}}{dt} = V_{medium} \cdot ksyn_{FXII} - V_{medium} \cdot kdeg_{FXII} \cdot \boldsymbol{FXII\_in\_plasma} - (k12 \cdot \boldsymbol{FXII\_in\_plasma} \cdot V_{medium} - k21 \cdot \boldsymbol{FXII} \cdot V_{proximal}) \quad \textbf{(E12)}$$

$$V_{medium}\frac{d\boldsymbol{FXIIa\_degraded}}{dt} = V_{medium} \cdot kdeg_{FXIIa} \cdot \boldsymbol{FXIIa\_in\_plasma} \quad \textbf{(E13)}$$

$$V_{medium}\frac{d\boldsymbol{FXIIa\_in\_plasma}}{dt} = -V_{medium} \cdot kdeg_{FXIIa} \cdot \boldsymbol{FXIIa\_in\_plasma} - V_{medium} \cdot (kon_{C1Inh\_FXIIa} \cdot \boldsymbol{C1Inh\_in\_plasma} \cdot \boldsymbol{FXIIa\_in\_plasma} - koff_{C1Inh\_FXIIa} \cdot \boldsymbol{C1Inh\_FXIIa\_in\_plasma}) - (k12 \cdot \boldsymbol{FXIIa\_in\_plasma} \cdot V_{medium} - k21 \cdot \boldsymbol{FXIIa} \cdot V_{proximal}) \quad \textbf{(E14)}$$

$$V_{medium}\frac{d\boldsymbol{HK2Chain\_degraded}}{dt} = V_{medium} \cdot kdeg_{cHMWK} \cdot \boldsymbol{HK2Chain\_in\_plasma} \quad \text{(E15)}$$

$$V_{medium}\frac{d\boldsymbol{HK2Chain\_in\_plasma}}{dt} = -V_{medium} \cdot (kon_{KAL\_HK2Chain} \cdot \boldsymbol{KAL\_in\_plasma} \cdot \boldsymbol{HK2Chain\_in\_plasma} - koff_{KAL\_HK2Chain} \cdot \boldsymbol{KAL\_HK2Chain\_in\_plasma}) - V_{medium} \cdot kdeg_{cHMWK} \cdot \boldsymbol{HK2Chain\_in\_plasma} \quad \text{(E16)}$$

$$V_{medium}\frac{d\boldsymbol{HMWK\_degraded}}{dt} = V_{medium} \cdot kdeg_{HMWK} \cdot \boldsymbol{HMWK\_in\_plasma} \quad \text{(E17)}$$

$$V_{medium}\frac{d\boldsymbol{HMWK\_in\_plasma}}{dt} = V_{medium} \cdot ksyn_{HMWK} - V_{medium} \cdot kdeg_{HMWK} \cdot \boldsymbol{HMWK\_in\_plasma} - V_{medium} \cdot (kon_{preKAL\_HMWK} \cdot \boldsymbol{preKAL\_in\_plasma} \cdot \boldsymbol{HMWK\_in\_plasma} - koff_{preKAL\_HMWK} \cdot \boldsymbol{preKAL\_HMWK\_in\_plasma}) - V_{medium} \cdot (kon_{KAL\_HMWK} \cdot \boldsymbol{KAL\_in\_plasma} \cdot \boldsymbol{HMWK\_in\_plasma} - koff_{KAL\_HMWK} \cdot \boldsymbol{KAL\_HMWK\_in\_plasma}) \quad \text{(E18)}$$

$$V_{medium}\frac{d\boldsymbol{KAL\_degraded}}{dt} = V_{medium} \cdot kdeg_{KAL} \cdot \boldsymbol{KAL\_in\_plasma} \quad \text{(E19)}$$

$$V_{medium}\frac{d\boldsymbol{KAL\_HK2Chain\_in\_plasma}}{dt} = -(k12 \cdot \boldsymbol{KAL\_HK2Chain\_in\_plasma} \cdot V_{medium} - k21 \cdot \boldsymbol{KAL\_HK2Chain} \cdot V_{proximal}) + V_{medium} \cdot (kon_{KAL\_HK2Chain} \cdot \boldsymbol{KAL\_in\_plasma} \cdot \boldsymbol{HK2Chain\_in\_plasma} - koff_{KAL\_HK2Chain} \cdot \boldsymbol{KAL\_HK2Chain\_in\_plasma}) \quad \text{(E20)}$$

$$V_{medium}\frac{d\boldsymbol{KAL\_HMWK\_in\_plasma}}{dt} = V_{medium}\cdot(kon_{KAL\_HMWK}\cdot\boldsymbol{KAL\_in\_plasma}\cdot\boldsymbol{HMWK\_in\_plasma} - koff_{KAL\_HMWK}\cdot\boldsymbol{KAL\_HMWK\_in\_plasma}) - V_{medium}\cdot(kon_{C1In\ \_KAL}\cdot\boldsymbol{C1Inh\_in\_plasma}\cdot\boldsymbol{KAL\_HMWK\_in\_plasma} - koff_{C1Inh\_KAL}\cdot\boldsymbol{C1Inh\_KAL\_HMWK\_in\_plasma}) - V_{medium}\cdot(kon_{KAL\_Lanadelumab}\cdot\boldsymbol{KAL\_HMWK\_in\_plasma}\cdot\boldsymbol{Lanadelumab\_in\_plasma} - koff_{KAL\_Lanadelumab}\cdot\boldsymbol{Lanadelumab\_KAL\_HMWK\_in\_plasma}) - (k12\cdot\boldsymbol{KAL\_HMWK\_in\_plasma}\cdot V_{medium} - k21\cdot\boldsymbol{KAL\_HMWK}\cdot V_{proximal}) \quad \textbf{(E21)}$$

$$V_{medium}\frac{d\boldsymbol{KAL\_in\_plasma}}{dt} = -V_{medium}\cdot kdeg_{KAL}\cdot\boldsymbol{KAL\_in\_plasma} - V_{medium}\cdot(kon_{KAL\_HMWK}\cdot\boldsymbol{KAL\_in\_plasma}\cdot\boldsymbol{HMWK\_in\_plasma} - koff_{KAL\_HMWK}\cdot\boldsymbol{KAL\_HMWK\_in\_plasma}) - V_{medium}\cdot(kon_{C1In\ \_KAL}\cdot\boldsymbol{C1Inh\_in\_plasma}\cdot\boldsymbol{KAL\_in\_plasma} - koff_{C1Inh\_KAL}\cdot\boldsymbol{C1Inh\_KAL\_in\_plasma}) - V_{medium}\cdot(kon_{KAL\_HK2Chain}\cdot\boldsymbol{KAL\_in\_plasma}\cdot\boldsymbol{HK2Chain\_in\_plasma} - koff_{KAL\_HK2Chain}\cdot\boldsymbol{KAL\_HK2Chain\_in\_plasma}) - V_{medium}\cdot(kon_{KAL\_Lanadelumab}\cdot\boldsymbol{KAL\_in\_plasma}\cdot\boldsymbol{Lanadelumab\_in\_plasma} - koff_{KAL\_Lanadelumab}\cdot\boldsymbol{Lanadelumab\_KAL\_in\_plasma}) \quad \textbf{(E22)}$$

$$V_{medium}\frac{d\boldsymbol{Lanadelumab\_KAL\_HMWK\_in\_plasma}}{dt} = V_{medium}\cdot(kon_{KAL\_Lanadelumab}\cdot\boldsymbol{KAL\_HMWK\_in\_plasma}\cdot\boldsymbol{Lanadelumab\_in\_plasma} - koff_{KAL\_Lanadelumab}\cdot\boldsymbol{Lanadelumab\_KAL\_HMWK\_in\_plasma}) - (k12\cdot\boldsymbol{Lanadelumab\_KAL\_HMWK\_in\_plasma}\cdot V_{medium} - k21\cdot\boldsymbol{Lanadelumab\_KAL\_HMWK}\cdot V_{proximal}) \quad \textbf{(E23)}$$

$$V_{medium}\frac{d\boldsymbol{Lanadelumab\_KAL\_in\_plasma}}{dt} = V_{medium}\cdot(kon_{KAL\_Lanadelumab}\cdot\boldsymbol{KAL\_in\_plasma}\cdot\boldsymbol{Lanadelumab\_in\_plasma} - koff_{KAL\_Lanadelumab}\cdot\boldsymbol{Lanadelumab\_KAL\_in\_plasma}) \quad \textbf{(E24)}$$

$$V_{medium}\frac{d\boldsymbol{preKAL\_degraded}}{dt} = V_{medium}\cdot kdeg_{preKAL}\cdot\boldsymbol{preKAL\_in\_plasma} \quad \textbf{(E25)}$$

$$V_{medium}\frac{d\boldsymbol{preKAL\_HMWK\_in\_plasma}}{dt} = V_{medium}\cdot(kon_{preKAL\_HMWK}\cdot\boldsymbol{preKAL\_in\_plasma}\cdot\boldsymbol{HMWK\_in\_plasma} - koff_{preKAL\_HMWK}\cdot\boldsymbol{preKAL\_HMWK\_in\_plasma}) - (k12\cdot\boldsymbol{preKAL\_HMWK\_in\_plasma}\cdot V_{medium} - k21\cdot\boldsymbol{preKAL\_HMWK}\cdot V_{proximal}) \quad \textbf{(E26)}$$

$$V_{medium}\frac{d\boldsymbol{preKAL\_in\_plasma}}{dt} = V_{medium}\cdot ksyn_{preKAL} - V_{medium}\cdot kdeg_{preKAL}\cdot\boldsymbol{preKAL\_in\_plasma} - V_{medium}\cdot(kon_{preKAL\_HMWK}\cdot\boldsymbol{preKAL\_in\_plasma}\cdot\boldsymbol{HMWK\_in\_plasma} - koff_{preKAL\_HMWK}\cdot\boldsymbol{preKAL\_HMWK\_in\_plasma}) \quad \textbf{(E27)}$$

In proximal space

$$V_{proximal}\frac{d\boldsymbol{BK}}{dt} = (k12_{BK}\cdot\boldsymbol{BK\_in\_plasma}\cdot V_{medium} - k21_{BK}\cdot\boldsymbol{BK}\cdot V_{proximal}) + (kcat_{HMWK\_cleavage}\cdot\boldsymbol{KAL\_HMWK\_gC1qR} - (kon_{BK\_BDK}\cdot\boldsymbol{BK}\cdot\boldsymbol{BDKRB2} - koff_{BK\_BDKRB2}\cdot\boldsymbol{BK\_BDKRB2}))\cdot Num\_to\_Conc\_converter\cdot V_{proximal} \quad \textbf{(E28)}$$

$$\frac{d\boldsymbol{BDKRB2}}{dt} = ksyn_{BDKRB2} - kdeg_{BDKRB2}\cdot\boldsymbol{BDKRB2} - (kon_{BK\_BDKRB2}\cdot\boldsymbol{BK}\cdot\boldsymbol{BDKRB2} - koff_{BK\_BDKRB2}\cdot\boldsymbol{BK\_BDKRB2}) \quad \textbf{(E29)}$$

$$\frac{d\boldsymbol{BDKRB2\_degraded}}{dt} = kdeg_{BDKRB2}\cdot\boldsymbol{BDKRB2} \quad \textbf{(E30)}$$

$$\frac{d\boldsymbol{BK\_BDKRB2}}{dt} = (kon_{BK\_BDKRB2} \cdot \boldsymbol{BK} \cdot \boldsymbol{BDKRB2} - koff_{BK\_BDKRB2} \cdot \boldsymbol{BK\_BDKRB2}) - kdeg_{BDKRB2} \cdot \boldsymbol{BK\_BDKRB2} \quad \textbf{(E31)}$$

$$\frac{d\boldsymbol{BK\_BDKRB2\_degraded}}{dt} = kdeg_{BDKRB2} \cdot \boldsymbol{BK\_BDKRB2} \quad \textbf{(E32)}$$

$$\frac{d\boldsymbol{C1Inh\_FXIIa\_gC1qR}}{dt} = (kon_{C1Inh\_FXIIa} \cdot \boldsymbol{C1Inh} \cdot \boldsymbol{FXIIa\_gC1qR} - koff_{C1Inh\_FXIIa} \cdot \boldsymbol{C1Inh\_FXIIa\_gC1qR}) + (kon_{FXIIa\_gC1qR} \cdot \boldsymbol{C1Inh\_FXIIa} \cdot \boldsymbol{gC1qR} - koff_{FXIIa\_gC1qR} \cdot \boldsymbol{C1Inh\_FXIIa\_gC1qR}) - kdeg_{gC1qR} \cdot \boldsymbol{C1Inh\_FXIIa\_gC1qR} \quad \textbf{(E33)}$$

$$\frac{d\boldsymbol{C1Inh\_FXIIa\_gC1qR\_degraded}}{dt} = kdeg_{gC1qR} \cdot \boldsymbol{C1Inh\_FXIIa\_gC1qR} \quad \textbf{(E34)}$$

$$\frac{d\boldsymbol{C1Inh\_KAL\_HMWK\_gC1qR}}{dt} = (kon_{C1Inh\_KAL} \cdot \boldsymbol{C1Inh} \cdot \boldsymbol{KAL\_HMWK\_gC1qR} - koff_{C1Inh\_KAL} \cdot \boldsymbol{C1Inh\_KAL\_HMWK\_gC1qR}) + (kon_{HMWK\_gC1} \cdot \boldsymbol{C1Inh\_KAL\_HMWK} \cdot \boldsymbol{gC1qR} - koff_{HMWK\_gC1qR} \cdot \boldsymbol{C1Inh\_KAL\_HMWK\_gC1qR}) - kdeg_{gC1qR} \cdot \boldsymbol{C1Inh\_KAL\_HMWK\_gC1qR} \quad \textbf{(E35)}$$

$$\frac{d\boldsymbol{C1Inh\_KAL\_HMWK\_gC1qR\_degraded}}{dt} = kdeg_{gC1qR} \cdot \boldsymbol{C1Inh\_KAL\_HMWK\_gC1qR} \quad \textbf{(E36)}$$

$$\frac{d\boldsymbol{FXII\_gC1qR}}{dt} = (kon_{FXII\_gC1qR} \cdot \boldsymbol{FXII} \cdot \boldsymbol{gC1qR} - koff_{FXII\_gC1qR} \cdot \boldsymbol{FXII\_gC1qR}) - Fold\_increase_{FXII\_AutoActivation} \cdot kcat_{FXII\_AutoActivation} \cdot \boldsymbol{FXII\_gC1qR} - kcat_{FXII\_AutoActivation} \cdot \boldsymbol{KAL\_HMWK\_gC1qR} \cdot \boldsymbol{FXII\_gC1qR} \cdot Num\_to\_Conc\_converter/(km_{FXII\_AutoActivation} + \boldsymbol{FXII\_gC1qR} \cdot Num\_to\_Conc\_converter) - kdeg_{gC1qR} \cdot \boldsymbol{FXII\_gC1qR} \quad \textbf{(E37)}$$

$$\frac{d\boldsymbol{FXII\_gC1qR\_degraded}}{dt} = kdeg_{gC1qR} \cdot \boldsymbol{FXII\_gC1qR} \quad \textbf{(E38)}$$

$$
\begin{aligned}
\frac{d\boldsymbol{FXIIa\_gC1qR}}{dt} = {} & Fold\_increase_{FXII\_AutoActivation} \cdot kcat_{FXII\_AutoActivation} \cdot \boldsymbol{FXII\_gC1qR} \\
& + kcat_{FXII\_AutoActivation} \cdot \boldsymbol{KAL\_HMWK\_gC1qR} \cdot \boldsymbol{FXII\_gC1qR} \\
& \cdot Num\_to\_Conc\_converter/(km_{FXII\_AutoActivation} + \boldsymbol{FXII\_gC1qR} \\
& \cdot Num\_to\_Conc\_converter) + (kon_{FXIIa\_gC1qR} \cdot \boldsymbol{FXIIa} \cdot \boldsymbol{gC1qR} \\
& - koff_{FXIIa\_gC1qR} \cdot \boldsymbol{FXIIa\_gC1qR}) - (kon_{C1Inh\_FXIIa} \cdot \boldsymbol{C1Inh} \\
& \cdot \boldsymbol{FXIIa\_gC1qR} - koff_{C1Inh\_FXIIa} \cdot \boldsymbol{C1Inh\_FXIIa\_gC1qR}) - kdeg_{gC1qR} \\
& \cdot \boldsymbol{FXIIa\_gC1qR}
\end{aligned}
\tag{E39}
$$

$$
\frac{d\boldsymbol{FXIIa\_gC1qR\_degraded}}{dt} = kdeg_{gC1qR} \cdot \boldsymbol{FXIIa\_gC1qR}
\tag{E40}
$$

$$
\begin{aligned}
\frac{d\boldsymbol{gC1qR}}{dt} = {} & ksyn_{gC1qR} - kdeg_{gC1qR} \cdot \boldsymbol{gC1qR} - (kon_{FXII\_gC1qR} \cdot \boldsymbol{FXII} \cdot \boldsymbol{gC1qR} \\
& - koff_{FXII\_gC1q} \cdot \boldsymbol{FXII\_gC1qR}) - (kon_{HMWK\_gC1qR} \cdot \boldsymbol{preKAL\_HMWK} \\
& \cdot \boldsymbol{gC1qR} - koff_{HMWK\_gC1qR} \cdot \boldsymbol{preKAL\_HMWK\_gC1qR}) - (kon_{FXIIa\_gC1} \\
& \cdot \boldsymbol{FXIIa} \cdot \boldsymbol{gC1qR} - koff_{FXIIa\_gC1qR} \cdot \boldsymbol{FXIIa\_gC1qR}) - (kon_{HMWK\_gC1qR} \\
& \cdot \boldsymbol{KAL\_HMWK} \cdot \boldsymbol{gC1qR} - koff_{HMWK\_gC1q} \cdot \boldsymbol{KAL\_HMWK\_gC1qR}) \\
& - (kon_{HK2Chain\_gC1qR} \cdot \boldsymbol{KAL\_HK2Chain} \cdot \boldsymbol{gC1qR} - koff_{HK2Chain\_gC1qR} \\
& \cdot \boldsymbol{KAL\_HK2Chain\_gC1qR}) - (kon_{FXIIa\_gC1qR} \cdot \boldsymbol{C1Inh\_FXIIa} \cdot \boldsymbol{gC1qR} \\
& - koff_{FXIIa\_gC1qR} \cdot \boldsymbol{C1Inh\_FXIIa\_gC1qR}) - (kon_{HMWK\_gC1qR} \\
& \cdot \boldsymbol{C1Inh\_KAL\_HMWK} \cdot \boldsymbol{gC1qR} - koff_{HMWK\_gC1qR} \\
& \cdot \boldsymbol{C1Inh\_KAL\_HMWK\_gC1qR}) - (kon_{HMWK\_gC1qR} \\
& \cdot \boldsymbol{Lanadelumab\_KAL\_HMWK} \cdot \boldsymbol{gC1qR} - koff_{HMWK\_gC1qR} \\
& \cdot \boldsymbol{Lanadelumab\_KAL\_HMWK\_gC1qR})
\end{aligned}
\tag{E41}
$$

$$
\frac{d\boldsymbol{gC1qR\_degraded}}{dt} = kdeg_{gC1qR} \cdot \boldsymbol{gC1qR}
\tag{E42}
$$

$$\frac{d\boldsymbol{KAL\_HK2Chain\_gC1qR}}{dt} = kcat_{HMWK\_cleavage} \cdot \boldsymbol{KAL\_HMWK\_gC1qR} + (kon_{HK2Chain\_gC1qR} \cdot \boldsymbol{KAL\_HK2Chain} \cdot \boldsymbol{gC1qR} - koff_{HK2Chain\_gC1qR} \cdot \boldsymbol{KAL\_HK2Chain\_gC1qR}) - kdeg_{gC1qR} \cdot \boldsymbol{KAL\_HK2Chain} \cdot \boldsymbol{gC1qR} \quad \text{(E43)}$$

$$\frac{d\boldsymbol{KAL\_HK2Chain\_gC1qR\_degraded}}{dt} = kdeg_{gC1qR} \cdot \boldsymbol{KAL\_HK2Chain\_gC1qR} \quad \text{(E44)}$$

$$\frac{d\boldsymbol{KAL\_HMWK\_gC1qR}}{dt} = kcat_{preKAL\_Activation} \cdot \boldsymbol{FXIIa\_gC1qR} \cdot \boldsymbol{preKAL\_HMWK\_gC1qR} \cdot Num\_to\_Conc\_converter/(km_{preKAL\_Activation} + \boldsymbol{preKAL\_HMWK\_gC1qR} \cdot Num\_to\_Conc\_converter) - kcat_{HMWK\_cleavage} \cdot \boldsymbol{KAL\_HMWK\_gC1qR} + (kon_{HMWK\_gC1qR} \cdot \boldsymbol{KAL\_HMWK} \cdot \boldsymbol{gC1qR} - koff_{HMWK\_gC1qR} \cdot \boldsymbol{KAL\_HMWK\_gC1qR}) - (kon_{C1Inh\_KAL} \cdot \boldsymbol{C1Inh} \cdot \boldsymbol{KAL\_HMWK\_gC1qR} - koff_{C1Inh\_KAL} \cdot \boldsymbol{C1Inh\_KAL\_HMWK\_gC1qR}) - kdeg_{gC1qR} \cdot \boldsymbol{KAL\_HMWK\_gC1qR} - (kon_{KAL\_Lanadelumab} \cdot \boldsymbol{KAL\_HMWK\_gC1qR} \cdot \boldsymbol{Lanadelumab} - koff_{KAL\_Lanadelumab} \cdot \boldsymbol{Lanadelumab\_KAL\_HMWK\_gC1qR}) \quad \text{(E45)}$$

$$\frac{d\boldsymbol{KAL\_HMWK\_gC1qR\_degraded}}{dt} = kdeg_{gC1qR} \cdot \boldsymbol{KAL\_HMWK\_gC1qR} \quad \text{(E46)}$$

$$\frac{d\boldsymbol{Lanadelumab\_KAL\_HMWK\_gC1qR}}{dt} = (kon_{KAL\_Lanadelumab} \cdot \boldsymbol{KAL\_HMWK\_gC1qR} \cdot \boldsymbol{Lanadelumab} - koff_{KAL\_Lanadelumab} \cdot \boldsymbol{Lanadelumab\_KAL\_HMWK\_gC1qR}) + (kon_{HMWK\_gC1qR} \cdot \boldsymbol{Lanadelumab\_KAL\_HMWK} \cdot \boldsymbol{gC1qR} - koff_{HMWK\_gC1qR} \cdot \boldsymbol{Lanadelumab\_KAL\_HMWK\_gC1qR}) - kdeg_{Lanadelumab\_KAL\_HMWK\_gC1qR} \cdot \boldsymbol{Lanadelumab\_KAL\_HMWK\_gC1qR} \quad \text{(E47)}$$

$$\frac{d\boldsymbol{Lanadelumba\_KAL\_HMWK\_gC1qR\_degraded}}{dt} = kdeg_{Lanadelumab\_KAL\_HMWK\_gC1qR} \cdot \boldsymbol{Lanadelumab\_KAL\_HMWK\_gC1qR} \quad \textbf{(E48)}$$

$$\frac{d\boldsymbol{preKAL\_HMWK\_gC1qR}}{dt} = (kon_{HMWK\_gC1} \cdot \boldsymbol{preKAL\_HMWK} \cdot \boldsymbol{gC1qR} - koff_{HMWK\_gC1qR} \cdot \boldsymbol{preKAL\_HMWK\_gC1qR}) - kcat_{preKAL\_Activation} \cdot \boldsymbol{FXIIa\_gC1qR} \cdot \boldsymbol{preKAL\_HMWK\_gC1qR} \cdot Num\_to\_Conc\_converter/(km_{preKAL\_Activation} + \boldsymbol{preKAL\_HMWK\_gC1qR} \cdot Num\_to\_Conc\_converter) - kdeg_{gC1qR} \cdot \boldsymbol{preKAL\_HMWK\_gC1qR} \quad \textbf{(E49)}$$

$$\frac{d\boldsymbol{preKAL\_HMWK\_gC1qR\_degraded}}{dt} = kdeg_{gC1qR} \cdot \boldsymbol{preKAL\_HMWK\_gC1qR} \quad \textbf{(E50)}$$

$$V_{proximal}\frac{d\boldsymbol{C1Inh}}{dt} = (k12 \cdot \boldsymbol{C1Inh\_in\_plasma} \cdot V_{medium} - k21 \cdot \boldsymbol{C1Inh} \cdot V_{proximal}) - (kon_{C1Inh\_FXIIa} \cdot \boldsymbol{C1Inh} \cdot \boldsymbol{FXIIa\_gC1qR} - koff_{C1Inh\_FXIIa} \cdot \boldsymbol{C1Inh\_FXIIa\_gC1qR} + kon_{C1Inh\_KAL} \cdot \boldsymbol{C1Inh} \cdot \boldsymbol{KAL\_HMWK\_gC1qR} - koff_{C1Inh\_KAL} \cdot \boldsymbol{C1Inh\_KAL\_HMWK\_gC1qR}) \cdot Num\_to\_Conc\_converter \cdot V_{proximal} \quad \textbf{(E51)}$$

$$V_{proximal}\frac{d\boldsymbol{C1Inh\_FXIIa}}{dt} = (k12 \cdot \boldsymbol{C1Inh\_FXIIa\_in\_plasma} \cdot V_{medium} - k21 \cdot \boldsymbol{C1Inh\_FXIIa} \cdot V_{proximal}) - (kon_{FXIIa\_gC1qR} \cdot \boldsymbol{C1Inh\_FXIIa} \cdot \boldsymbol{gC1qR} - koff_{FXIIa\_gC1qR} \cdot \boldsymbol{C1Inh\_FXIIa\_gC1qR}) \cdot Num\_to\_Conc\_converter \cdot V_{proximal} \quad \textbf{(E52)}$$

$$V_{proximal}\frac{d\boldsymbol{C1Inh\_KAL\_HMWK}}{dt} = (k12\cdot \boldsymbol{C1Inh\_KAL\_HMWK\_in\_plasma}\cdot V_{medium} - k21 \cdot \boldsymbol{C1Inh\_KAL\_HMWK}\cdot V_{proximal}) - (kon_{HMWK\_gC1qR}\cdot \boldsymbol{C1Inh\_KAL\_HMWK} \cdot \boldsymbol{gC1qR} - koff_{HMWK\_gC1qR}\cdot \boldsymbol{C1Inh\_KAL\_HMWK\_gC1qR}) \cdot Num\_to\_Conc\_converter\cdot V_{proximal} \quad \textbf{(E53)}$$

$$V_{proximal}\frac{d\boldsymbol{FXII}}{dt} = (k12\cdot \boldsymbol{FXII\_in\_plasma}\cdot V_{medium} - k21\cdot \boldsymbol{FXII}\cdot V_{proximal}) - (kon_{FXII\_gC1}\ \cdot \boldsymbol{FXII}\cdot \boldsymbol{gC1qR} - koff_{FXII\_gC1q}\ \cdot \boldsymbol{FXII\_gC1qR}) \cdot Num\_to\_Conc\_converter\cdot V_{proximal} \quad \textbf{(E54)}$$

$$V_{proximal}\frac{d\boldsymbol{FXIIa}}{dt} = (k12\cdot \boldsymbol{FXIIa\_in\_plasma}\cdot V_{medium} - k21\cdot \boldsymbol{FXIIa}\cdot V_{proximal}) - (kon_{FXIIa\_gC1qR}\cdot \boldsymbol{FXIIa}\cdot \boldsymbol{gC1qR} - koff_{FXIIa\_gC1}\ \cdot \boldsymbol{FXIIa\_gC1qR}) \cdot Num\_to\_Conc\_converter\cdot V_{proximal} \quad \textbf{(E55)}$$

$$V_{proximal}\frac{d\boldsymbol{KAL\_HK2Chain}}{dt} = (k12\cdot \boldsymbol{KAL\_HK2Chain\_in\_plasma}\cdot V_{medium} - k21\cdot \boldsymbol{KAL\_HK2Chain} \cdot V_{proximal}) - (kon_{HK2Chain\_gC1qR}\cdot \boldsymbol{KAL\_HK2Chain}\cdot \boldsymbol{gC1qR} - koff_{HK2Chain\_gC1qR}\cdot \boldsymbol{KAL\_HK2Chain\_gC1qR})\cdot Num\_to\_Conc\_converter \cdot V_{proximal} \quad \textbf{(E56)}$$

$$V_{proximal}\frac{d\boldsymbol{KAL\_HMWK}}{dt} = (k12\cdot \boldsymbol{KAL\_HMWK\_in\_plasma}\cdot V_{medium} - k21\cdot \boldsymbol{KAL\_HMWK} \cdot V_{proximal}) - (kon_{HMWK\_gC1qR}\cdot \boldsymbol{KAL\_HMWK}\cdot \boldsymbol{gC1qR} - koff_{HMWK\_gC1qR} \cdot \boldsymbol{KAL\_HMWK\_gC1qR})\cdot Num\_to\_Conc\_converter\cdot V_{proximal} \quad \textbf{(E57)}$$

$$V_{proximal}\frac{d\boldsymbol{Lanadelumab\_KAL\_HMWK}}{dt} = (k12 \cdot \boldsymbol{Lanadelumab\_KAL\_HMWK\_in\_plasma} \cdot V_{medium} - k21 \cdot \boldsymbol{Lanadelumab\_KAL\_HMWK} \cdot V_{proximal}) - (kon_{HMWK\_gC1qR} \cdot \boldsymbol{Lanadelumab\_KAL\_HMWK} \cdot \boldsymbol{gC1qR} - koff_{HMWK\_gC1} \cdot \boldsymbol{Lanadelumab\_KAL\_HMWK\_gC1qR}) \cdot Num\_to\_Conc\_converter \cdot V_{proximal} \quad \textbf{(E58)}$$

$$V_{proximal}\frac{d\boldsymbol{preKAL\_HMWK}}{dt} = (k12 \cdot \boldsymbol{preKAL\_HMWK\_in\_plasma} \cdot V_{medium} - k21 \cdot \boldsymbol{preKAL\_HMWK} \cdot V_{proximal}) - (kon_{HMWK\_gC1qR} \cdot \boldsymbol{preKAL\_HMWK} \cdot \boldsymbol{gC1qR} - koff_{HMWK\_gC1qR} \cdot \boldsymbol{preKAL\_HMWK\_gC1qR}) \cdot Num\_to\_Conc\_converter \cdot V_{proximal} \quad \textbf{(E59)}$$

BDKR-B2 B2 receptor of bradykinin receptor family, *BK* bradykinin, *C1-INH* C1 esterase inhibitor, *C1q* complement component 1, *FXII* factor XII, *FXIIa* activated factor XII, *gC1q-R* cofactors complement protein C1q, *HMWK* high molecular weight kininogen, K_{off} dissociation constant, K_{on} association constant, *KAL* kallikrein, *preKAL* prekallikrein, V_{medium} per endothelial cell-based vascular volume, $V_{proximal}$ per endothelial cell-based proximal space volume

Table S6 List of model parameters

Parameter	Description	Unit	Value	Source
In vascular space				
Kd_KAL_HK2Chain	K_d for "KAL_in_plasma + HK2Chain_in_plasma ↔ KAL_HK2Chain_in_plasma"	nM	72	Kenniston et al [24]
Kd_KAL_HMWK	K_d for "KAL_in_plasma + HMWK_in_plasma ↔ KAL_HMWK_in_plasma"	nM	15	Bock et al [5]
Kd_preKAL_HMWK	K_d for "preKAL_in_plasma + HMWK_in_plasma ↔ preKAL_HMWK_in_plasma"	nM	12	Bock et al [5]
kdeg_BK	Degradation rate for bradykinin	1/h	55.452	Décarie et al (1996) [27]
kdeg_Bound_C1Inh	Degradation rate for bound C1-INH	1/h	13.863	Estimated from Jensen et al [28]
kdeg_C1Inh	Degradation rate for C1-INH	1/h	0.0165	Weidmann et al [22]
kdeg_cHMWK	Degradation rate for cHMWK	1/h	0.0619	Calibrated with guidance from typical range in proteins
kdeg_FXII	Degradation rate for FXII	1/h	0.0116	Weidmann et al [22]
kdeg_FXIIa	Degradation rate for FXIIa	1/h	8.318	Assumed same as kallikrein
kdeg_HMWK	Degradation rate for HMWK	1/h	0.0044	Weidmann et al [22]
kdeg_KAL	Degradation rate for kallikrein	1/h	8.318	Cumming et al [29]

kdeg_preKAL	Degradation rate for prekallikrein	1/h	0.0289	Calculated using a half-life of 24 hours (Labcorp.com)
koff_KAL_HK2Chain	k_{off} for kallikrein and cHMWK binding event	1/h	318.816	Calculated from K_d and k_{on} of the corresponding reaction
koff_KAL_HMWK	k_{off} for kallikrein and HMWK binding event	1/h	66.42	Calculated from K_d and k_{on} of the corresponding reaction
koff_preKAL_HMWK	k_{off} for prekallikrein & HMWK binding event	1/h	53.136	Calculated from K_d and k_{on} of the corresponding reaction
kon_KAL_HK2Chain	k_{on} for "KAL_in_plasma + HK2Chain_in_plasma ↔ KAL_HK2Chain_in_plasma"	1/(M×h)	4.428	Assumed 10× kon_HMWK_gC1qR, due to lower steric constraint
kon_KAL_HMWK	k_{on} for "KAL_in_plasma + HMWK_in_plasma ↔ KAL_HMWK_in_plasma"	1/(M×h)	4.428	Assumed 10× kon_HMWK_gC1qR, due to lower steric constraint
kon_preKAL_HMWK	k_{on} for "preKAL_in_plasma + HMWK_in_plasma ↔ preKAL_HMWK_in_plasma"	1/(M×h)	4.428	Assumed 10× kon_HMWK_gC1qR, due to lower steric constraint
ksyn_C1Inh	Synthesis rate for C1-INH	nM/h	11.883 HAE 39.608 Healthy	Calibrated with guidance from steady-state level data in Table S2

ksyn_FXII	Synthesis rate for FXII	nM/h	10.83	Calibrated with guidance from steady-state level data in Table S2
ksyn_HMWK	Synthesis rate for HMWK	nM/h	39.933	Calibrated with guidance from steady-state level data in Table S2
ksyn_preKAL	Synthesis rate for prekallikrein	nM/h	41.589	Calibrated with guidance from steady-state level data in Table S2
Vmedium	Per endothelial cell based plasma volume	L	1.23E-12	Estimated from Shah and Betts [30] and Bianconi et al [31]
In proximal space				
BDKRB2_per_Cell_SS	The number of bradykinin B2 receptors per cell at steady state	-	100,000	Estimated from Paquet et al [14]
gC1qR_per_Cell_SS	The number of gC1q receptors per cell at steady state	-	100,000	Mahdi et al [9], assumed limited by the CK1 cofactor
kcat_FXII_Activation	k_{cat} for FXII activation (substrate: FXII_gC1qR; enzyme: KAL_HMWK_gC1qR; product: FXIIa_gC1qR)	1/h	15	Calibrated with guidance from steady-state level data in Table S2

kcat_FXII_AutoActivation	k_{cat} for FXII autoactivation (substrate: FXII_gC1qR; enzyme: FXII_gC1qR; product: FXIIa_gC1qR)	1/h	0.0475	Calibrated with guidance from steady-state level data in Table S2
kcat_HMWK_cleavage	k_{cat} for cleavage of HMWK	1/h	394.7	Calibrated with guidance from steady-state level data in Table S2
kcat_preKAL_Activation	k_{cat} for prekallikrein activation (substrate: preKAL_HMWK_gC1qR; enzyme: FXIIa_gC1qR; product: KAL_HMWK_gC1qR)	1/h	18	Calibrated with guidance from steady-state level data in Table S2
Kd_BK_BDKRB2	k_d for "BK + BDKRB2 ↔ BK_BDKRB2"	nM	0.5	Paquet et al [14]
Kd_FXII_gC1qR	k_d for "FXII + gC1qR ↔ FXII_gC1qR"	nM	144	Reddigari et al [7]
Kd_FXIIa_gC1qR	k_d for "FXIIa + gC1qR ↔ FXIIa_gC1qR"	nM	144	Assumed same as Kd_FXII_gC1qR
Kd_HK2Chain_gC1qR	k_d for "KAL_HK2Chain + gC1qR ↔ KAL_HK2Chain_gC1qR"	nM	10.35	Assumed same as kon_HMWK_gC1qR
Kd_HMWK_gC1qR	k_d for "preKAL_HMWK + gC1qR ↔ preKAL_HMWK_gC1qR"	nM	10.35	Fernando et al [32]

kdeg_BDKRB2	Degradation rate for bradykinin B2 receptors	1/h	0.3466	Used typical degradation rate for receptors, as reported by Morelon and Dautry-Varsat [33]
kdeg_gC1qR	Degradation rate for gC1q receptor complex on endothelial cell surface	1/h	0.3466	Used typical degradation rate for receptors, as reported by Morelon and Dautry-Varsat [33]
kdeg_Lanadelumab_KAL_HMWK_gC1qR	Degradation rate for lanadelumab bound with KAL_HMWK_gC1qR receptor complex	1/h	0.3466	Assumed same as the degradation rate for unbound receptors
Km_FXII_Activation	K_m for FXII activation (substrate: FXII_gC1qR; enzyme: KAL_HMWK_gC1qR; product: FXIIa_gC1qR)	nM	510	Tankersley and Finlayson [26]
Km_FXII_AutoActivation	K_m for FXII autoactivation (substrate: FXII_gC1qR; enzyme: FXII_gC1qR; product: FXIIa_gC1qR)	nM	110	Bernardo et al [34]
Km_preKAL_Activation	K_m for prekallikrein activation (substrate: preKAL_HMWK_gC1qR; enzyme: FXIIa_gC1qR;	nM	91	Tankersley and Finlayson [26]

	product: KAL_HMWK_gC1qR)			
koff_BK_BDKRB2	k_{off} for bradykinin & bradykinin B2 receptor binding event	1/h	18	Calculated from K_d and k_{on} of the corresponding reaction
koff_FXII_gC1qR	k_{off} for FXII and gC1q receptor binding event	1/h	63.763	Calculated from K_d and k_{on} of the corresponding reaction
koff_FXIIa_gC1qR	k_{off} for FXIIa & gC1q receptor binding event	1/h	63.763	Calculated from K_d and k_{on} of the corresponding reaction
koff_HK2Chain_gC1qR	k_{off} for cHMWK & and gC1q receptor binding event	1/h	4.583	Calculated from K_d and k_{on} of the corresponding reaction
koff_HMWK_gC1qR	k_{off} for HMWK & gC1q receptor binding event	1/h	4.583	Calculated from K_d and k_{on} of the corresponding reaction
kon_BK_BDKRB2	k_{on} for "BK + BDKRB2 ↔ BK_BDKRB2"	1/(M×h)	36	Assumed fast binding for bradykinin due to its low molecular weight
kon_FXII_gC1qR	k_{on} for "FXII + gC1qR ↔ FXII_gC1qR"	1/(M×h)	0.4428	Assumed same as kon_HMWK_gC1qR
kon_FXIIa_gC1qR	k_{on} for "FXIIa + gC1qR ↔ FXIIa_gC1qR"	1/(M×h)	0.4428	Assumed same as kon_HMWK_gC1qR

kon_HK2Chain_gC1qR	k_{on} for "KAL_HK2Chain + gC1qR ↔ KAL_HK2Chain_gC1qR"	1/(M×h)	0.4428	Assumed same as kon_HMWK_gC1qR
kon_HMWK_gC1qR	k_{on} for "preKAL_HMWK + gC1qR ↔ preKAL_HMWK_gC1qR"	1/(M×h)	0.4428	Pixley et al [6]
ksyn_BDKRB2	Synthesis rate for bradykinin B2 receptors	number/cell/h	34657.359	Estimated from steady-state level data from Paquet et al [14]
ksyn_gC1qR	Synthesis rate for gC1q receptor complex on endothelial cell surface	number/cell/h	34657.359	Estimated from steady-state level data from Mahdi et al [9]
Vproximal	Proximal space volume near cell surface for each endothelial cell	L	8.00E-15	20 nm (height) × 400 um^2 (surface area) [35, 36]
In vascular and proximal space				
Kd_C1Inh_FXIIa	k_d for "C1Inh_in_plasma + FXIIa_in_plasma ↔ C1Inh_FXIIa_in_plasma" and "C1Inh + FXIIa_gC1qR ↔ C1Inh_FXIIa_gC1qR"	nM	1720	Estimated from Biacore data by Björkqvist et al [37]
Kd_C1Inh_KAL	k_d for "C1Inh_in_plasma + KAL_in_plasma ↔ C1Inh_KAL_in_plasma", "C1Inh_in_plasma +	nM	150	Estimated from immunosorbent assay data by Harpel et al [25]

	KAL_HMWK_in_plasma ↔ C1Inh_KAL_HMWK_in_plasma", and "C1Inh + KAL_HMWK_gC1qR ↔ C1Inh_KAL_HMWK_gC1qR"			
Kd_KAL_Lanadelumab	k_d for "KAL_in_plasma + Lanadelumab_in_plasma ↔ Lanadelumab_KAL_in_plasma", "KAL_HMWK_in_plasma + Lanadelumab_in_plasma ↔ Lanadelumab_KAL_HMWK_in_plasma", and "KAL_HMWK_gC1qR + Lanadelumab ↔ Lanadelumab_KAL_HMWK_gC1qR"	nM	0.12	Shire measurement, Kenniston et al [24]
koff_C1Inh_FXIIa	k_{off} for "C1Inh_in_plasma + FXIIa_in_plasma ↔ C1Inh_FXIIa_in_plasma" and "C1Inh + FXIIa_gC1qR ↔ C1Inh_FXIIa_gC1qR"	1/h	229.104	Calculated from K_d and k_{on} of the corresponding reaction
koff_C1Inh_KAL	k_{off} for "C1Inh_in_plasma + KAL_in_plasma ↔ C1Inh_KAL_in_plasma",	1/h	9.18	Calculated from K_d and k_{on} of the corresponding reaction

	"C1Inh_in_plasma + KAL_HMWK_in_plasma ↔ C1Inh_KAL_HMWK_in_plasma", and "C1Inh + KAL_HMWK_gC1qR ↔ C1Inh_KAL_HMWK_gC1qR"			
koff_KAL_Lanadelumab	k_{off} for "KAL_in_plasma + Lanadelumab_in_plasma ↔ Lanadelumab_KAL_in_plasma", "KAL_HMWK_in_plasma + Lanadelumab_in_plasma ↔ Lanadelumab_KAL_HMWK_in_plasma", and "KAL_HMWK_gC1qR + Lanadelumab ↔ Lanadelumab_KAL_HMWK_gC1qR"	1/h	1.452	Calculated from K_d and k_{on} of the corresponding reaction
kon_C1Inh_FXIIa	k_{on} for "C1Inh_in_plasma + FXIIa_in_plasma ↔ C1Inh_FXIIa_in_plasma" and "C1Inh + FXIIa_gC1qR ↔ C1Inh_FXIIa_gC1qR"	1/(nM× h)	0.1332	Drouet et al [38]
kon_C1Inh_KAL	k_{on} for "C1Inh_in_plasma + KAL_in_plasma ↔	1/(nM× h)	0.0612	Drouet et al [38]

	C1Inh_KAL_in_plasma", "C1Inh_in_plasma + KAL_HMWK_in_plasma ↔ C1Inh_KAL_HMWK_in_plasma", and "C1Inh + KAL_HMWK_gC1qR ↔ C1Inh_KAL_HMWK_gC1qR"			
kon_KAL_Lanadelumab	k_{on} for "KAL_in_plasma + Lanadelumab_in_plasma ↔ Lanadelumab_KAL_in_plasma", "KAL_HMWK_in_plasma + Lanadelumab_in_plasma ↔ Lanadelumab_KAL_HMWK_in_plasma", and "KAL_HMWK_gC1qR + Lanadelumab ↔ Lanadelumab_KAL_HMWK_gC1qR"	1/(nM× h)	12.096	Shire measurement
Exchange between vascular and proximal space				
k12	Species exchange rate from plasma to proximal space	1/h	0.2341	Assumed based circulation time from Shah and Betts [30]

k21	Species exchange rate from proximal space to plasma	1/h	36	Assumed based circulation time from Shah and Betts [30]
k12_BK	Bradykinin exchange rate from plasma to proximal space	1/h	7.0244	Calibrated to match BK level in Table S2
k21_BK	Bradykinin exchange rate from proximal space to plasma	1/h	1080	Calibrated to match BK level in Table S2

BDKR-B2 B2 receptor of bradykinin receptor family, *BK* bradykinin, *C1-INH* C1 esterase inhibitor, *C1q* complement component 1, *cHMWK* cleaved high molecular weight kininogen, *CK1* cytokeratin 1, *FXII* factor XII, *FXIIa* activated factor XII, *gC1q-R* cofactors complement protein C1q, *HAE* hereditary angioedema, *HK2* human kallikrein 2, *HMWK* high molecular weight kininogen, *KAL* kallikrein, k_{cat} turnover number, K_d binding affinity, K_{off} dissociation constant, K_{on} association constant, K_{syn} synthesis rate, *preKAL* prekallikrein

Table S7 Model assumptions

1)	The CK1 cofactor with the lowest level of expression is the limiting number for formation of the gCq1-R/CK1/uPAR receptor complex in surface contact system activation. An apparent number of sites and affinity to different contact factors are used.
2)	The effects of Zn^{+2} dependency on binding affinities and the endothelial cell prekallikrein activator (PRCP) are not explicitly included in the model and their effects are assumed to be implicitly reflected in the model parameters.
3)	The exchange rate between the vascular space and the proximal space is assumed to be of the same order as the vascular volume circulation time (approximately 100 seconds [30]).
4)	Various triggers of HAE attacks (e.g. stress, physical trauma, a surgical or a dental procedure, infection, hormonal changes, mechanical pressure) are assumed to lead to a systematic perturbation that autoactivates the kinin-kallikrein pathway in the contact activation system.
5)	The rise in the pain is triggered by an acute attack and the duration of the attack is represented by the time period over which the level of FXII autoactivation remains elevated.
6)	The inhibitory activity of lanadelumab on kallikrein in plasma is the same as that of kallikrein bound to the surface, and the inhibitory activity of C1-INH on kallikrein and FXIIa in plasma is the same as on the surface.

C1-INH C1 esterase inhibitor, *CK1* cytokeratin 1, *C1q* complement component 1, *FXII* factor XII, *FXIIa* activated factor XII, *gC1q-R* cofactors complement protein C1q, *HAE* hereditary angioedema, *PRCP* prolylcarboxypeptidase, *uPAR* urokinase plasminogen activating receptor

Fig. S1 Overview of the process for development of the HAE QSP model. *C1-INH* C1 esterase inhibitor, *cHMWK* cleaved high molecular weight kininogen, *HAE* hereditary angioedema, *KKS* kallikrein-kinin system, *PD* pharmacodynamics, *PK* pharmacokinetics, *QSP* quantitative systems pharmacology

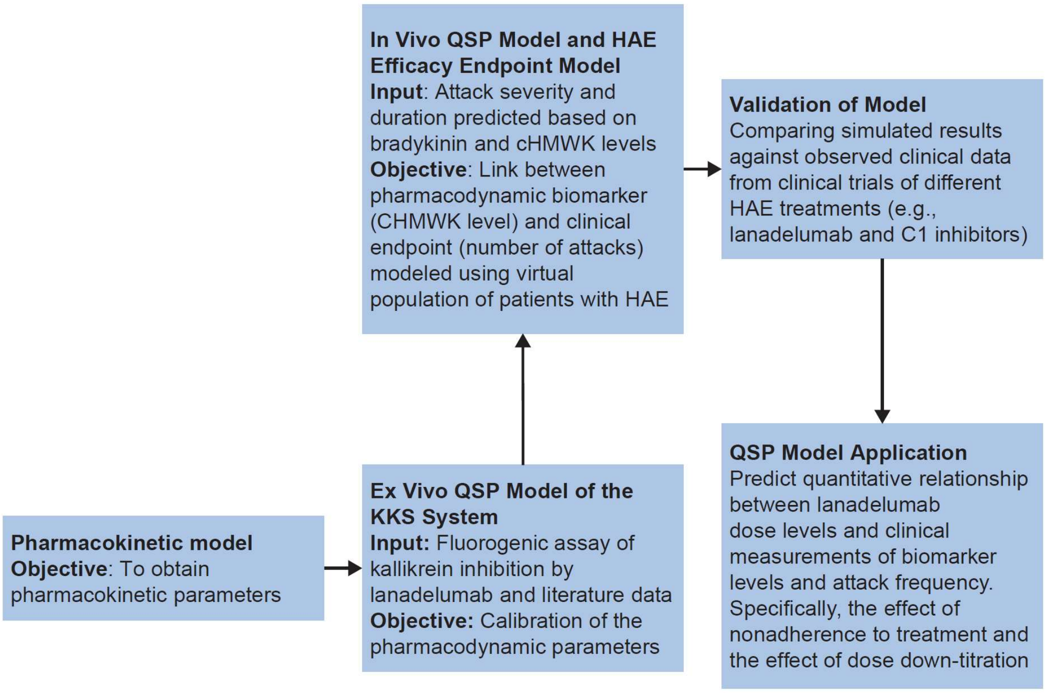

Fig. S2 Ex vivo assay measuring the inhibition of proteolytic activity of kallikrein by lanadelumab. The assay utilizes a synthetic peptide substrate (Pro-Phe-Arg-aminomethyl coumarin) that exhibits increased fluorescence upon proteolysis catalyzed by kallikrein. A key assumption is that the fluorescence readout can be directly related to the level of free kallikrein. *FXIIa* activated factor XII, *SC* subcutaneous

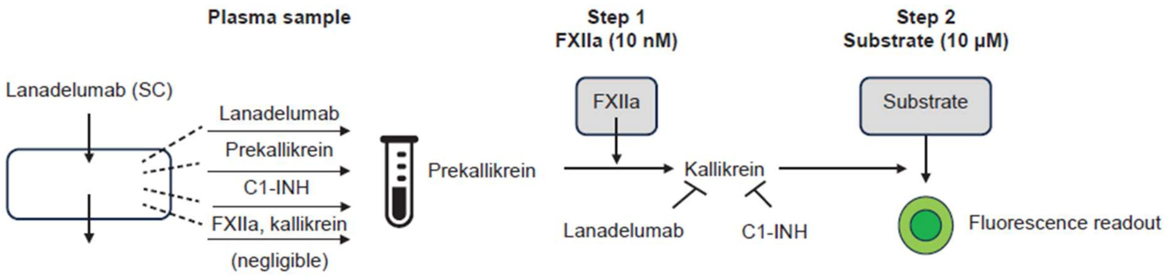

Fig. S3 Map of the KKS in HAE and pharmacodynamic interactions. The model reflects the biology of the KKS in HAE involving the essential contact factor proteins, such as FXII/FXIIa, prekallikrein/kallikrein and HMWK, which are activated on the endothelial cell surface to release the vasoactive peptide bradykinin. The pathway is a cascade of activation and cleavage reactions associated with these proteins and their complexes in plasma and bound to receptor complexes on the cell surface. *BDKR-B2* B2 receptor of bradykinin receptor family, *BK* bradykinin*,* *C1-INH* C1 esterase inhibitor, *FXII* factor XII, *FXIIa* activated factor XII, *HAE* hereditary angioedema, *HMWK* high molecular weight kininogen, *KAL* kallikrein, *mAb* monoclonal antibody, *KKS* kallikrein-kinin system, *preKAL* prekallikrein

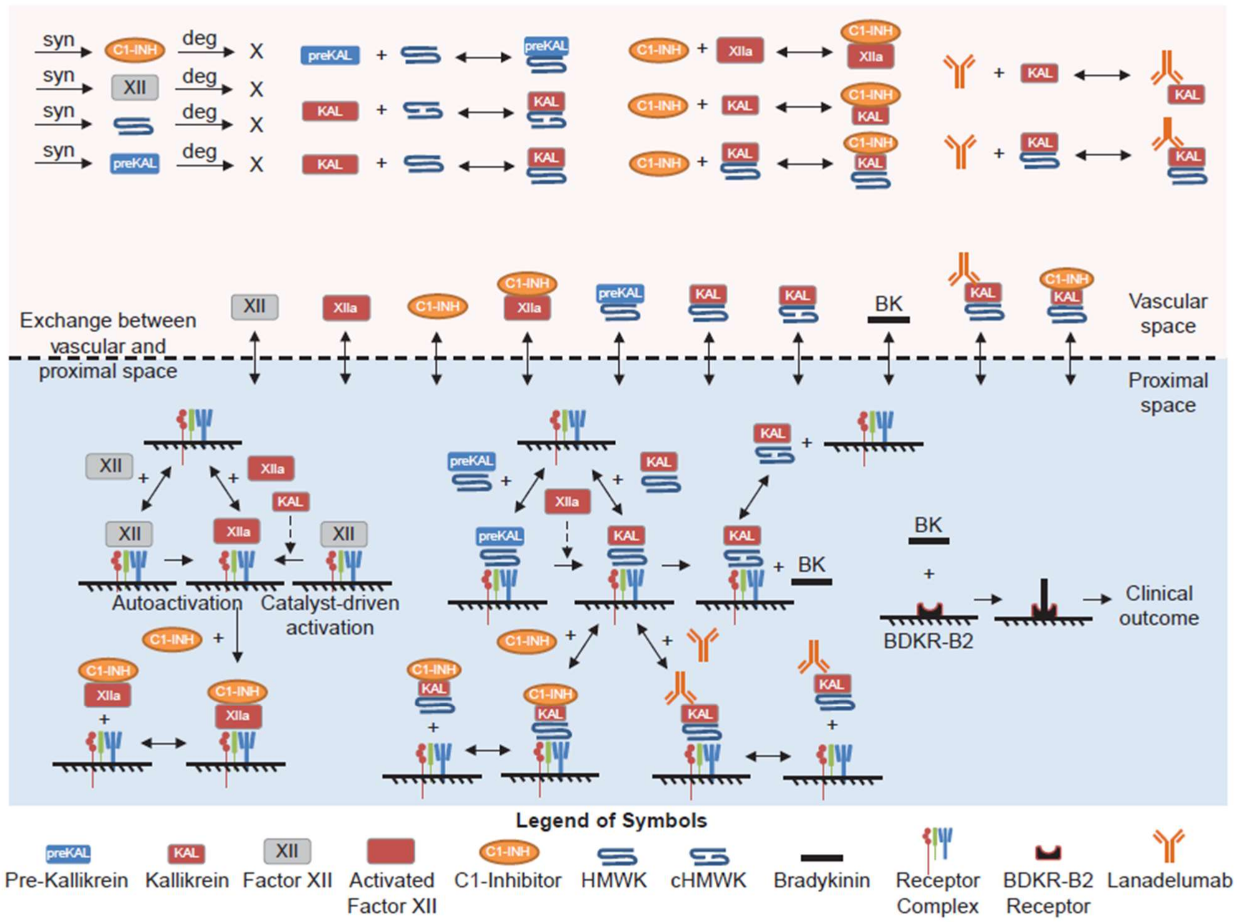

Fig. S4 Inhibition of plasma kallikrein by lanadelumab. Output from the simulation of lanadelumab treatment (shaded band) were compared to data from study phase 1 [15] (a) and phase 1b [16] (b) at different dose levels of lanadelumab. The prekallikrein range corresponds to the range reported by Madsen et al [1]. Negative % inhibition values arise when excess ex vivo KKS activation occurs in pre-dose samples during blood collection and processing to plasma, resulting in a lower enzymatic rate in the pre-dose sample [% inhibition = (1 – post-dose rate / pre-dose rate) x 100]. *preKAL* prekallikrein

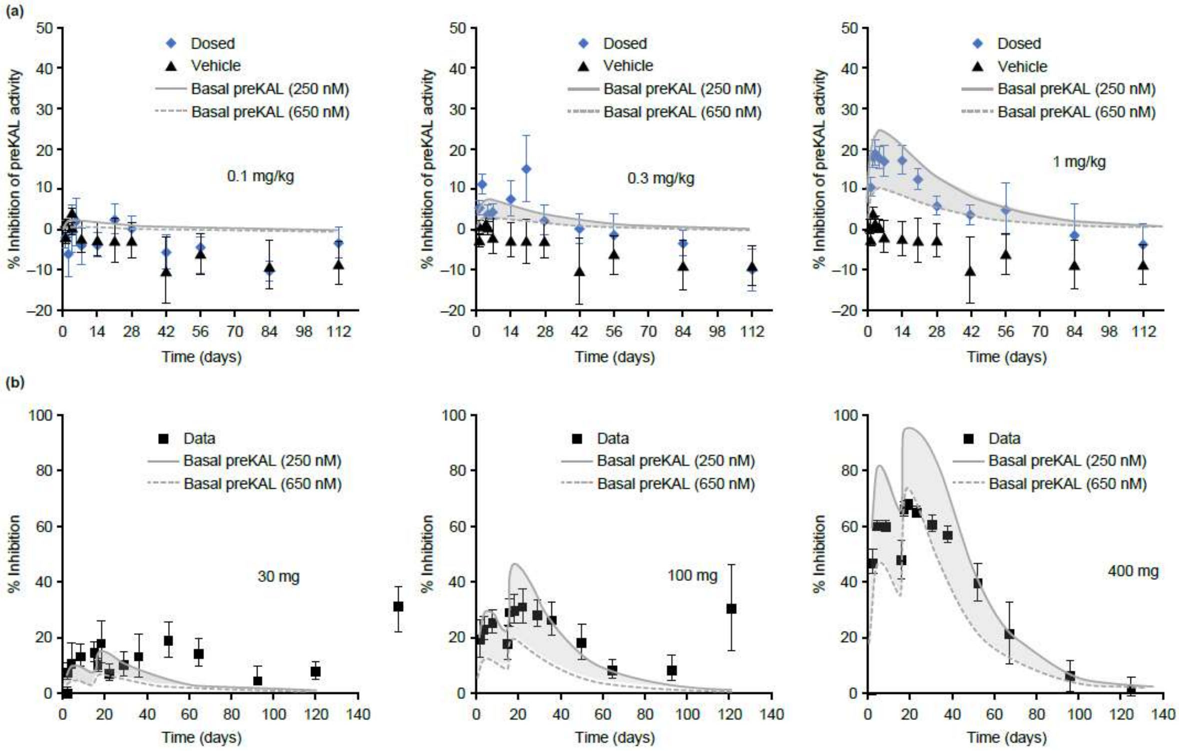

Fig. S5 Comparison of steady-state protein levels predicted by the KKS model to data from the literature. Error bars represent the range of levels reported in the literature. Free preKAL% refers to the percentage of prekallikrein in free form (i.e. not bound to HMWK) relative to total prekallikrein. %cHMWK is the percentage of cHMWK relative to the total of cHMWK + HMWK. *BK* bradykinin, *C1-INH* C1 esterase inhibitor, *cHMWK* cleaved high molecular weight kininogen, *HAE* hereditary angioedema, *HC* healthy controls, *HMWK* high molecular weight kininogen, *KKS* kallikrein-kinin system, *preKAL* prekallikrein

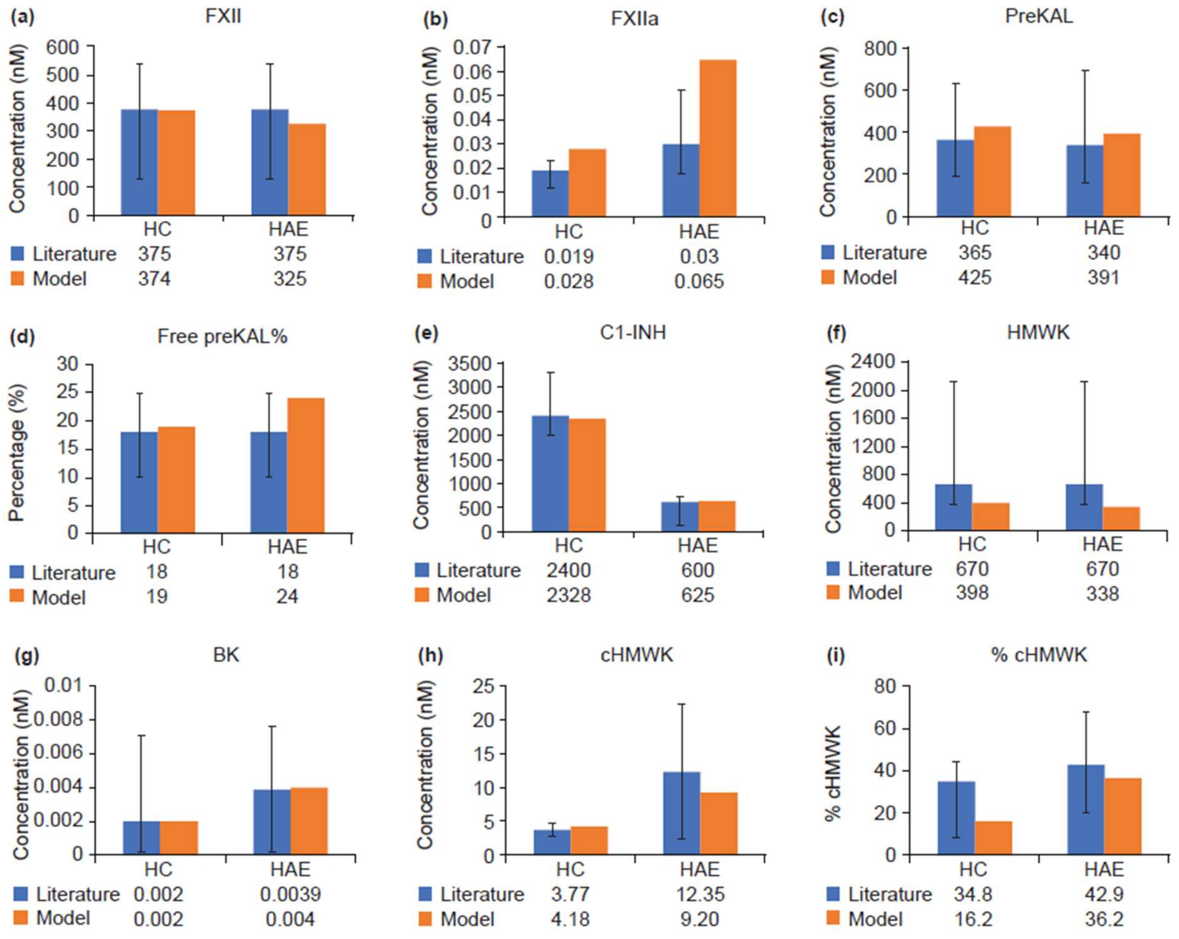

Fig. S6 Simulated PK profiles were compared with mean concentrations of functional C1-INH protein in HAE patients treated with (a) a single IV dose of 1000 IU C1-INH or (b) 1000 IU C1-INH twice/week for 12 weeks [19]. *C1-INH* C1 esterase inhibitor, *HAE* hereditary angioedema, *IV* intravenous, *PK* pharmacokinetics

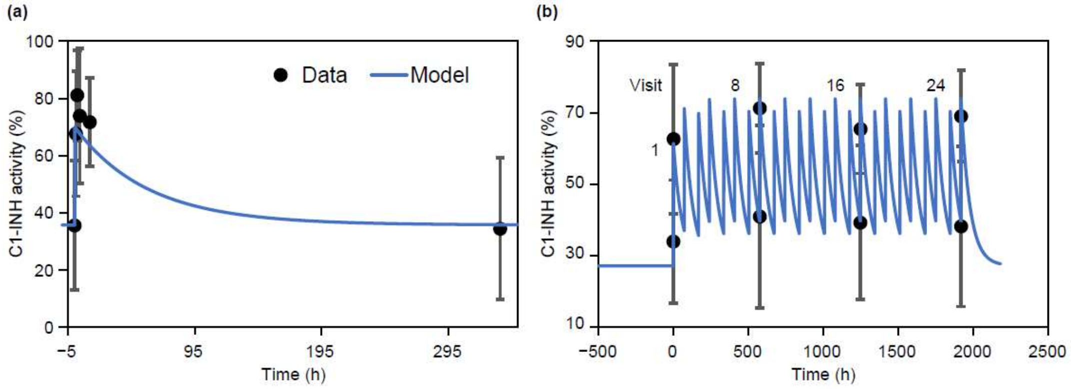

Fig. S7 Predicted impact of down-titration for patients with high baseline attack rate (6 attacks/month). (a) Bradykinin levels and (b) attack rate with lanadelumab Q2W dosing for 6 months followed by Q4W dosing for 6 months. Arrows indicate the following: (1) first 300 mg Q2W dose (week 0); (2) first 300 mg Q4W dose (week 24); and (3) last 300 mg Q4W dose (week 44). Bradykinin graph shows mean (black line) and 5% and 95% CI (shaded area). Dotted line shows bradykinin threshold (systemic concentration) for risk of attack (0.02 nM). Month –1 indicates baseline. *HAE* hereditary angioedema, *Q2W* every 2 weeks, *Q4W* every 4 weeks

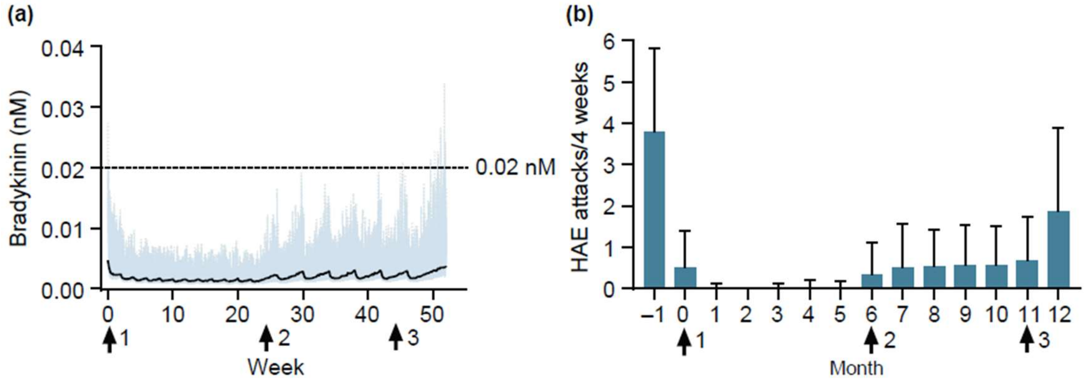

References

1. Madsen DE, Sidelmann JJ, Biltoft D, Gram J, Hansen S (2015) C1-inhibitor polymers activate the FXII-dependent kallikrein–kinin system: Implication for a role in hereditary angioedema. Biochim Biophys Acta 1850:1336-1342. https://doi.org/10.1016/j.bbagen.2015.03.005
2. Mandle RJ, Colman RW, Kaplan AP (1976) Identification of prekallikrein and high-molecular-weight kininogen as a complex in human plasma. Proc Natl Acad Sci U S A 73:4179-4183. https://doi.org/10.1073/pnas.73.11.417
3. Colman RW, Schmaier AH (1997) Contact system: a vascular biology modulator with anticoagulant, profibrinolytic, antiadhesive, and proinflammatory attributes. Blood 90:3819-3843
4. Shariat-Madar Z, Mahdi F, Schmaier AH (2002) Assembly and activation of the plasma kallikrein/kinin system: a new interpretation. International immunopharmacology 2:1841-1849
5. Bock P, Shore J, Tans G, Griffin J (1985) Protein-protein interactions in contact activation of blood coagulation. Binding of high molecular weight kininogen and the 5-(iodoacetamido) fluorescein-labeled kininogen light chain to prekallikrein, kallikrein, and the separated kallikrein heavy and light chains. J Biol Chem 260:12434-12443
6. Pixley RA, Espinola RG, Berhane Ghebrehiwet KJ, Kao A, Bdeir K, Cines DB, Colman RW (2011) Interaction of high-molecular-weight kininogen with endothelial cell binding proteins suPAR, gC1qR and cytokeratin 1 determined by surface plasmon resonance (BiaCore). Thromb Haemost 105:1053-1059. https://doi.org/10.1160/TH10-09-0591
7. Reddigari S, Shibayama Y, Brunnee T, Kaplan A (1993) Human Hageman factor (factor XII) and high molecular weight kininogen compete for the same binding site on human umbilical vein endothelial cells. Journal of Biological Chemistry 268:11982-11987
8. Joseph K, Shibayama Y, Nakazawa Y, Peerschke EI, Ghebrehiwet B, Kaplan AP (1999) Interaction of factor XII and high molecular weight kininogen with cytokeratin 1 and gC1qR of vascular endothelial cells and with aggregated Aβ protein of Alzheimer's disease. Immunopharmacology 43:203-210. https://doi.org/10.1016/s0162-3109(99)00136-8
9. Mahdi F, Shariat-Madar Z, Todd III RF, Figueroa CD, Schmaier AH (2001) Expression and colocalization of cytokeratin 1 and urokinase plasminogen activator receptor on endothelial cells. Blood 97:2342-2350. https://doi.org/10.1182/blood.v97.8.2342
10. Peerschke E, Smyth SS, Teng EI, Dalzell M, Ghebrehiwet B (1996) Human umbilical vein endothelial cells possess binding sites for the globular domain of C1q. J Immunol 157:4154-4158
11. Barnathan ES, Kuo A, Rosenfeld L, Kariko K, Leski M, Robbiati F, Nolli M, Henkin J, Cines DB (1990) Interaction of single-chain urokinase-type plasminogen activator with human endothelial cells. J Biol Chem 265:2865-2872
12. Kaplan AP, Ghebrehiwet B (2010) The plasma bradykinin-forming pathways and its interrelationships with complement. Mol Immunol 47:2161-2169. https://doi.org/10.1016/j.molimm.2010.05.010
13. Erdös EG, Yang H (1970) Kininases. In: Bradykinin, kallidin and kallikrein. Springer, Berlin, Germany, pp 289-323

14. Paquet JL, Luccarini JM, Fouchet C, Defrêne E, Loillier B, Robert C, Bélichard P, Cremers B, Pruneau D (1999) Pharmacological characterization of the bradykinin B2 receptor: inter-species variability and dissociation between binding and functional responses. Br J Pharmacol 126:1083-1090. https://doi.org/10.1038/sj.bjp.0702403
15. Chyung Y, Vince B, Iarrobino R, Sexton D, Kenniston J, Faucette R, TenHoor C, Stolz LE, Stevens C, Biedenkapp J (2014) A phase 1 study investigating DX-2930 in healthy subjects. Ann Allergy Asthma Immunol 113:460-466.e462
16. Banerji A, Busse P, Shennak M, Lumry W, Davis-Lorton M, Wedner HJ, Jacobs J, Baker J, Bernstein JA, Lockey R (2017) Inhibiting plasma kallikrein for hereditary angioedema prophylaxis. N Engl J Med 376:717-728. https://doi.org/10.1056/NEJMoa1605767
17. Banerji A, Riedl MA, Bernstein JA, Cicardi M, Longhurst HJ, Zuraw BL, Busse PJ, Anderson J, Magerl M, Martinez-Saguer I (2018) Effect of lanadelumab compared with placebo on prevention of hereditary angioedema attacks: a randomized clinical trial. JAMA 320:2108-2121. https://doi.org/10.1001/jama.2018.16773
18. Banerji A, Bernstein JA, Johnston DT, Lumry WR, Magerl M, Maurer M, Martinez-Saguer I, Zanichelli A, Hao J, Inhaber N, Yu M, Riedl MA (2021) Long-term prevention of hereditary angioedema attacks with lanadelumab: the HELP OLE Study. Allergy 77:979-990. https://doi.org/10.1111/all.15011
19. Zuraw BL, Busse PJ, White M, Jacobs J, Lumry W, Baker J, Craig T, Grant JA, Hurewitz D, Bielory L (2010) Nanofiltered C1 inhibitor concentrate for treatment of hereditary angioedema. N Engl J Med 363:513-522. https://doi.org/10.1056/NEJMoa0805538
20. Riedl MA, Hurewitz DS, Levy R, Busse PJ, Fitts D, Kalfus I (2012) Nanofiltered C1 esterase inhibitor (human) for the treatment of acute attacks of hereditary angioedema: an open-label trial. Annals of Allergy, Asthma & Immunology 108:49-53. https://doi.org/10.1016/j.anai.2011.10.017
21. ClinicalTrials.gov (2021) Open-label c1 esterase inhibitor (C1INH-nf) for the prevention of acute hereditary angioedema (HAE) attacks (CHANGE 3). https://clinicaltrials.gov/ct2/show/results/NCT00462709. Accessed 2 February 2022
22. Weidmann H, Heikaus L, Long AT, Naudin C, Schlüter H, Renné T (2017) The plasma contact system, a protease cascade at the nexus of inflammation, coagulation and immunity. Biochim Biophys Acta Mol Cell Res 1864:2118-2127. https://doi.org/10.1016/j.bbamcr.2017.07.009
23. Cicardi M, Igarashi T, Rosen FS, Davis AE, 3rd (1987) Molecular basis for the deficiency of complement 1 inhibitor in type I hereditary angioneurotic edema. J Clin Invest 79:698-702. https://doi.org/10.1172/jci112873
24. Kenniston JA, Faucette RR, Martik D, Comeau SR, Lindberg AP, Kopacz KJ, Conley GP, Chen J, Viswanathan M, Kastrapeli N (2014) Inhibition of plasma kallikrein by a highly specific active site blocking antibody. J Biol Chem 289:23596-23608. https://doi.org/10.1074/jbc.M114.569061
25. Harpel PC, Lewin MF, Kaplan AP (1985) Distribution of plasma kallikrein between C-1 inactivator and alpha 2-macroglobulin in plasma utilizing a new assay for alpha 2-macroglobulin-kallikrein complexes. J Biol Chem 260:4257-4263
26. Tankersley DL, Finlayson JS (1984) Kinetics of activation and autoactivation of human factor XII. Biochemistry 23:273-279. https://doi.org/10.1021/bi00297a016
27. Décarie A, Raymond P, Gervais N, Couture R, Adam A (1996) Serum interspecies differences in metabolic pathways of bradykinin and [des-Arg9]BK: influence of

enalaprilat. Am J Physiol 271:H1340-H1347. https://doi.org/10.1152/ajpheart.1996.271.4.H1340
28. Jensen PE, Humle Jørgensen S, Datta P, Sørensen PS (2004) Significantly increased fractions of transformed to total alpha2-macroglobulin concentrations in plasma from patients with multiple sclerosis. Biochim Biophys Acta 1690:203-207. https://doi.org/10.1016/j.bbadis.2004.06.010
29. Cumming A, Robertson C, Jeffrey S, Robson J, Ledingham I (1984) The plasma kallikrein kinin system in severely ill and traumatised patients. Arch Emerg Med 1:135-142. https://doi.org/10.1136/emj.1.3.135
30. Shah DK, Betts AM (2012) Towards a platform PBPK model to characterize the plasma and tissue disposition of monoclonal antibodies in preclinical species and human. Journal of pharmacokinetics and pharmacodynamics 39:67-86
31. Bianconi E, Piovesan A, Facchin F, Beraudi A, Casadei R, Frabetti F, Vitale L, Pelleri MC, Tassani S, Piva F, Perez-Amodio S, Strippoli P, Canaider S (2013) An estimation of the number of cells in the human body. Ann Hum Biol 40:463-471. https://doi.org/10.3109/03014460.2013.807878
32. Fernando LP, Natesan S, Joseph K, Kaplan AP (2003) High molecular weight kininogen and factor XII binding to endothelial cells and astrocytes. Thromb Haemost 90:787-795. https://doi.org/10.1160/TH03-04-0231
33. Morelon E, Dautry-Varsat A (1998) Endocytosis of the common cytokine receptor gammac chain. Identification of sequences involved in internalization and degradation. J Biol Chem 273:22044-22051. https://doi.org/10.1074/jbc.273.34.22044
34. Bernardo MM, Day DE, Olson ST, Shore JD (1993) Surface-independent acceleration of factor XII activation by zinc ions. I. Kinetic characterization of the metal ion rate enhancement. J Biol Chem 268:12468-12476
35. Garipcan B, Maenz S, Pham T, Settmacher U, D. JK, Zanow J, Bossert J (2010) Image analysis of endothelial microstructure and endothelial cell dimensions of human arteries – a preliminary study. Adv Eng Mat 13:B54-B57
36. Félétou M (2011) The endothelium: part 1: multiple functions of the endothelial cells—focus on endothelium-derived vasoactive mediators. Morgan & Claypool Life Sciences, San Rafael, California
37. Björkqvist J, de Maat S, Lewandrowski U, Di Gennaro A, Oschatz C, Schönig K, Nöthen MM, Drouet C, Braley H, Nolte MW, Sickmann A, Panousis C, Maas C, Renné T (2015) Defective glycosylation of coagulation factor XII underlies hereditary angioedema type III. J Clin Invest 125:3132-3146. https://doi.org/10.1172/JCI77139
38. Drouet C, Ponard D, Ghannam A (2018) Chapter 23: C1 inhibitor. In: The Complement FactsBook. 2nd edn. Academic Press, Cambridge, Massachusetts, pp 241-249
